# Immunotherapy in Elderly Patients Affected by Non-Small Cell Lung Cancer: A Narrative Review

**DOI:** 10.3390/jcm12051833

**Published:** 2023-02-24

**Authors:** Michele Montrone, Gerardo Rosati, Vito Longo, Annamaria Catino, Raffaella Massafra, Annalisa Nardone, Francesco Pesola, Elisabetta Sara Montagna, Ilaria Marech, Pamela Pizzutilo, Domenico Galetta

**Affiliations:** 1 Medical Thoracic Oncology Unit, IRCCS Istituto Tumori “Giovanni Paolo II”, 70124 Bari, Italy; 2 Medical Oncology Unit, “San Carlo” Hospital, 85100 Potenza, Italy; 3 Health Physics Unit, IRCCS Istituto Tumori “Giovanni Paolo II”, 70124 Bari, Italy; 4 Radiotherapy Unit, IRCCS Istituto Tumori “Giovanni Paolo II”, 70124 Bari, Italy

**Keywords:** non-small cell lung cancer, immunotherapy, immune check point inhibitors

## Abstract

Lung cancer is the leading cause of cancer-related deaths worldwide. Non-small cell lung cancer (NSCLC) accounts for approximately 80% of all lung cancers, and most NSCLC is diagnosed in the advanced stage. The advent of immune check point inhibitors (ICIs) changed the therapeutic scenario both in metastatic disease (in first and subsequent lines) and earlier settings. Comorbidities, reduced organ function, cognitive deterioration, and social impairment give reasons for a greater probability of adverse events, making the treatment of elderly patients challenging. The reduced toxicity of ICIs compared to standard chemotherapy makes this approach attractive in this population. The effectiveness of ICIs varies according to age, and patients older than 75 years may benefit less than younger patients. This may be related to the so-called immunosenescence, a phenomenon that refers to the reduced activity of immunity with older age. Elders are often under-represented in clinical trials, even if they are a large part of the patients in a clinical practice. In this review, we aim to explore the biological aspects of immunosenescence and to report and analyze the most relevant and recent literature findings on the role of immunotherapy in elderly patients with NSCLC.

## 1. Introduction

Lung cancer is one of the most frequently occurring cancers: it is the third most frequent cancer and the first cause of cancer death worldwide (both sexes and all ages) [1]. More than 60% of cancer patients are aged 65 or older [2]. About 47% of lung cancer diagnoses performed in the US before 2003 occurred in patients aged ≥70 years, and 14% of the cases were diagnosed in patients aged 80 years or more [3]. According to the most recent statistics in the US (SEER 22 2015–2019), the median age at diagnosis is 71 years and the peak incidence is in the age group between 65 and 74 years; more than 70% of patients are 65 years old or older, 36.2% of cases are diagnosed in patient aged 75 or older, and 9.4% in those aged ≥84 years [4]. Therefore, mortality is higher among patients aged 65–74 (32.7%), it is 29.1% among patients aged 75–84, and the median age at death is 72 years [4].

Non-small cell lung cancer (NSCLC) constitutes between 80% and 85% of all lung cancers [5].

Given these statistics, there is an urgent need to optimize the treatment of NSCLC in patients aged over 70 years old. Factors, such as higher risk of comorbidities and deteriorations in physical, organ, and cognitive functions [6,7], together with pharmacokinetic differences between young and elderly patients [8] and different social support (such as the need to depend on a caregiver or the difficulty in accessing a cancer center), make the treatment of older adults quite challenging.

Older patients often are undertreated, due to the clinician’s opinion of reduced tolerability, not supported by adequate geriatric assessment. In clinical practice, elderly patients are treated mostly with palliative rather than curative intent, often with personalized chemotherapy doses and schedules.

Elderly patients are under-represented in clinical trials, despite the high prevalence of cancer and NSCLC over the age of 70. In a FDA analysis of cancer patients enrolled in trials supporting registration from 2005–2015, only 24% of patients were 70 years or older, and only the 12% were older than 75 [9]. An analysis of 248 phase III trials of systemic therapy for advanced NSCLC between 1980 and 2010 demonstrated that among the 100 most cited trials, 33% excluded elderly patients in their design (age exclusion ranged from >65 to >75 years of age); the average reported patient median age in these trials was 60.9 years, and the average age for trials that did not exclude elderly patients was 61 [10].

The advent of immunotherapy represented a huge step forward in the treatment of NSCLC, especially for non-oncogene addicted patients, both in the first and in subsequent lines. It has been shown to be more effective and tolerated than standard chemotherapy, with an improved quality of life (QoL). Immune check point inhibitors (ICIs) were therefore very welcomed among oncologists, also for the possibility of treating frail patients, including elderly patients.

However, the effectiveness of ICIs may vary according to age, and it has been suggested that patients older than 75 years may benefit less than younger patients. This may be related to the so-called immunosenescence, a phenomenon which refers to the reduced activity of immunity with older age.

In this narrative review, we aim to report and analyze the most relevant and recent literature findings on the role of immunotherapy in elderly patients with NSCLC, with reference mainly to pivotal clinical trials in advanced disease, with a look also to locally advanced and early stages, given the recent findings in this setting in phase 3 clinical trials.

## 2. Definition of Older Patient

A unique definition of an elderly patient in the field of oncology has not yet been established to date. The World Health Organization (WHO) describes ageing populations as those over aged 60 years, stating that policy should be framed to improve the functional ability of all older people [11]. However, this definition may not be appropriate in oncology, as more than 60% of cancer patients are aged 65 or older, and 16% of those older than 65 years have a history of cancer [2]. The age of 65 years has been often identified as a cut-off to define the elderly population in the subgroup analyses of clinical trials, and it is generally considered as the cut-off for the chronologic definition of an older adult and eligibility for Medicare benefits. On the other hand, the age of 70 years has been identified as a threshold to identify and screen elders in geriatric assessment dedicated to older cancer patients [12]. Biological aging must be distinguished from chronological aging; life expectancy, vulnerability and fragility, comorbidities, and drug therapies must be taken into account when assessing the age of the cancer patient to be treated.

The Japan Lung Cancer Society guideline defines elderly patients with lung cancer as those “75 years or older” [13].

Due to the lack of an unambiguous definition, we will evaluate the data coming from the main clinical trials in the field of immune-oncology in NSCLC patients, considering the age-specified subgroup analyses. We will also discuss data from retrospective analyses and a few studies dedicated to elderly patients.

## 3. Immunosenescence

The aging process of the immune system has been brilliantly described by Ferrara et al. [14].

With advancing age, both adaptive and innate immune responses are involved in a remodeling process defined as immunosenescence, and this could account for a reduced immune system response to cancer cells or other stimuli [14].

The main organs involved in immunity (thymus, spleen, lymph nodes, and bone marrow) undergo a progressive volume reduction and involution. Further, chronic antigenic stimulation contributes to this phenomenon by a reduction in the number of naïve lymphocytes due to a progressive conversion from naïve to memory cells, coupled with a reduction of lymphocytes de novo production. Therefore, there is a reduction of the antigenic diversity of immune cells associated with a detrimental immune response [14]. Aging is characterized by a lower proliferative activity of T cells and other immune cells, whereas their functions are not necessarily decreased [15,16,17]. This is coupled with up-regulation of immune suppressive signals [18,19].

CD28 is a major co-stimulatory receptor responsible for the optimal antigen-mediated T-cell activation, proliferation, and survival; with aging, the accumulation of CD28-T cells, especially in the CD8 subset, is observed; these cells exhibit reduced antigen receptor diversity, defective antigen-induced proliferation, and a shorter replicative lifespan, while showing enhanced cytotoxicity and regulatory functions [20]. Further, CD27 costimulatory molecule is reduced with aging [14]. This is coupled with an increase of cytotoxic associate molecule CD57 on T cells [21], leading to an activation of the cytotoxic arm of the immune response [14]. Moreover, senescent T cells secrete high levels of proinflammatory cytokines [17,22]. Besides the up-regulation of CD57 and the down-regulation of CD27 and CD28, the expression of the co-inhibitory killer-cell lectin-like receptor G1 (KLRG1) is observed [23]; KLRG1 identifies T cells in humans that are capable of secreting cytokines, but that fail to proliferate after stimulation [23]. Thus, CD28-CD27-CD57+KLRG1+ may represent reliable phenotypic biomarkers for T-cell terminal differentiation and senescence in the periphery [14].

Interestingly, a reduction of CD28+CD57-CD8+ and an increase of CD28-CD57+CD8+ T cells has been observed in patients with advanced lung cancer, accounting for a reduced proliferative capacity and dysfunctional antitumor activity [14,24].

Looking at B-cell compartment, the number of B cells decreases faster with aging, while the level of circulating immunoglobulins increases; actually, the level of high-specificity immunoglobulis is decreased, whereas the production of auto-antibodies and pro-inflammatory cytokines is enhanced, determining a low-grade state of chronic inflammation defined as “inflammaging” [14,22,25].

Further, innate immune response is affected by aging. Antigen-presenting cells show impaired proteasome functions, determining a reduced activity of antigen processing [26] coupled with a reduced secretion of pro-inflammatory cytokines [27] and reduced expression of Toll-Like Receptors [28], resulting in impaired immune activation of both innate and adaptive responses [14].

Phagocytic activity of polymorphonulcear cells and NK cytolitic activity is reduced in elders [29,30]. Further, the number of peripheral blood dendritic cells could be reduced by aging [31], as well as the antitumor activity of in vitro IFN-γ stimulated macrophages [32].

All these aging-related changes of innate and adaptive immunity could account for a higher risk of cancer onset and progression, as well as for a decreased response to ICIs in elders, together with an increased risk of immune-relate adverse events (irAEs) due to a higher concentration of pro-inflammatory cytokines and autoantibodies [14].

Another intriguing aspect is the role of gut microbiota in the immunosenescence process, with the evidence that the maintenance of youthful gut microbiota architecture could delay immunosenescence: probiotics and prebiotics may down-regulate the proinflammatory response and improve the innate immune function in the elderly [14]. In addition, syringaresinol (extract from Panax ginseng) enhances the Firmicutes/Bacteroidetes ratio that is associated with reinforced gut integrity and diminished inflammation in middle-aged mice. These modifications of gut microbiota are associated with an increase of naïve T cells, reduction of regulatory T cells, and restoration of T-cell effector functions [33].

The study of immunosenescence processes represents, therefore, an intriguing field of investigation, with the aim of reverting the aging of immunity and potentially increasing responses to ICIs, together with a reduction of irAEs [14].

## 4. PD-1/PD-L1 Axis Inhibition in the Second-Line Setting

The efficacy and safety of ICIs in NSCLC was first evaluated in the second-line setting, at the failure of platinum-based chemotherapy (Table 1).

Nivolumab was the first-in-class ICI introduced in clinical practice and demonstrated its efficacy over Docetaxel after the failure of platinum-based doublet chemotherapy in two phase 3 trials [34,35].

In the CheckMate 017, 131 patients with squamous NSCLC were randomly assigned to receive Nivolumab and 129 to Docetaxel; Nivolumab was associated with better survival, with a 41% of reduction of risk of death (HR 0.59; 95% CI, 0.44–0.79; *p* < 0.001). Patients aged 65 to 74 years old were 91 (33%), and among them was observed a 44% reduction of risk of death (HR 0.56; 95% CI, 0.34–0.91). Patients aged 75 years old or more were only 29 (11%), and they did not benefit from Nivolumab (HR 1.85; 95% CI, 0.76–4.51) [34].

The CheckMate 057 trial enrolled 582 patients with nonsquamous NSCLC; 287 were randomly assessed to receive Nivolumab, and 268 received Docetaxel [35]. Nivolumab demonstrated to improve survival, with a 27% of reduction of risk of death (HR 0.73; 95% CI, 0.59–0.89; *p* 0.002). In this trial, 200 (34%) patients were aged between 65 and 75 years old, and 43 (7%) were aged over 75 years. Among patients aged ≥65 years, a 37% reduction of the risk of death was observed in the nivolumab group, compared to the Docetaxel group: HR 0.63 (95% CI, 0.45–0.89). In the over-75-years-old group, a 10% reduction in the risk of death was noted with Nivolumab, as compared with docetaxel: HR 0.90 (95% CI, 0.43–1.87) [35].

The efficacy of Nivolumab versus Docetaxel in previously treated patients with NSCLC was confirmed also in the pooled analyses of Checkmate 017 and Checkmate 057 at 2, 3, 4, and 5 years [36,52,53,54]. Overall, 427 patients were randomly assigned to Nivolumab and 427 to Docetaxel. In the pooled 5-year analysis, Nivolumab continued to show a benefit compared to Docetaxel: HR for OS: 0.68 (95% CI, 0.59–0.78), with 13.4% of patients alive at 5 years (95% CI, 10.4 to 16.9) with Nivolumab versus 2.6% (95% CI, 1.4–4.5) with docetaxel [36]. Nivolumab showed an advantage in OS after 5 years in patients under 65 years of age (HR 0.66) and in those over 65 years (HR 0.71), but this benefit is lost when considering people aged 75 years or more (HR 1.19), even though they were only 72 patients [36].

The phase 2 trial KEYNOTE-010 enrolled previously treated NSCLC patients with a PD-L1 tumor proportion score (TPS) of at least 1% [55]. A total of 1034 patients were randomly assigned (1:1:1) to receive Pembrolizumab 2 mg/kg, Pembrolizumab 10 mg/kg, or Docetaxel, resulting in improved survival with Pembrolizumab in the total population: the HR for Pembrolizumab 2 mg/kg versus Docetaxel was 0.71 (95% CI 0.58–0.88; *p* = 0.0008), and the HR for Pembrolizumab 10 mg/kg versus Docetaxel was 0.61 (95% CI 0.49–0.75; *p* < 0.0001). Patients aged ≥65 years were 429 (41%); in this subgroup, HR for survival was 0.76 (95% CI 0.57–1.02) [55]. Further, the updated 5-year survival analysis for the intent-to-treat (ITT) population of KEYNOTE-010 confirmed the survival advantage for Pembrolizumab: mOS of 11.8 months (10.4–13.1) versus 8.4 months (7.6–9.5) in the PD-L1 TPS ≥ 1% group, with a HR of 0.70 (0.61–0.80) [37]. The benefit for patients aged ≥65 years persisted also at the 5-year follow-up, with a reduction of risk of death of 20%: HR 0.80 (0.60–1.01) [37].

Atezolizumab was compared to Docetaxel in pretreated NSCLC patients in the phase 2 trial POPLAR, which enrolled 287 patients, demonstrating a significant improvement in OS [56]. In the phase 3 trial OAK, 850 patients included in the primary analysis representing the ITT population were recruited and randomly assigned to Atezolizumab (425) and Docetaxel (425) [38]. Median OS was confirmed to be better with Atezolizumab: HR 0.73 (95% CI, 0.62–0.87), *p* 0.0003. Patients aged 65 years old or more were 47% of the overall population; the 45% in the Atezolizumab arm and the 49% in the Docetaxel arm. Further, in this subgroup, mOS was better with Atezolizumab: mOS 14.1 versus 9.2 months, HR 0.66 (CI 95%, 0.52–0.83) [38]. The final results from the randomized phase 2 POPLAR and phase 3 OAK clinical trials, after a median follow-up of 48.6 months for POPLAR and 47.7 months for OAK, confirmed the survival benefit with Atezolizumab versus Docetaxel in patients with previously treated NSCLC, regardless of PD-L1 expression and histology [57]. Considering long-term survivors (≥4 years) in POPLAR patients aged 65 years old or more were the 7% of the same population in the ITT in the Atezolizumab arm and 4% in the Docetaxel arm, in OAK they were the 6% in the Atezolizumab arm and 4% in the Docetaxel arm [57].

## 5. PD-1/PD-L1 Axis Inhibition in the First-Line Setting

Several phase 3 trials compared single-agent ICI versus chemotherapy, demonstrating the superiority of immunotherapy in patients with PD-L1 positive and PD-L1 TPS ≥50%. Table 1 summarizes the main studies that have proven to be practice changing in this setting.

The first phase III study showing the superiority of immunotherapy over platinum-based chemotherapy in the first-line setting was the KEYNOTE-024 trial [58]. Pembrolizumab was compared to standard chemotherapy in previously untreated patients with a PD-L1 TPS ≥ 50% (both squamous and non-squamous histology) and wild type (WT) for EGFR and ALK, demonstrating superiority in terms of PFS and OS, with a well-known safety profile consistent with all ICIs. Median age was 64.5 years in the Pembrolizumab group and 66 years in the chemotherapy group. The PFS benefit was observed in both age subgroups: HR of 0.61 (95% CI, 0.40–0.92) in the under-65-years group and HR of 0.45 (95% CI 0.29–0.60) in those ≥65 years [58]. HR for OS was 0.60 (95% CI, 0.38–0.96) in patients under 65 years and 0.64 (95% CI, 0.42–0.98) in those ≥65 years [39]. At the updated analysis (5 years follow-up), Pembrolizumab continued to show remarkable activity, with mOS of 26.3 months (95% CI, 18.3–40.4) versus 13.4 months (95% CI, 9.4–18.3 months) in the chemotherapy group, HR 0.62 (95% CI, 0.48–0.81). OS rates at 5 years were 31.9% (95% CI, 24.5–39.5) in the Pembrolizumab group and 16.3% (95% CI, 10.6–23.0) in the chemotherapy group. Any new safety signal was noted with extended follow-up [59].

Similarly, the KEYNOTE-042 trial, which enrolled patients with PD-L1 TPS ≥ 1%, showed improved survival with Pembrolizumab over chemotherapy in the first-line setting in the overall population (HR 0.11 [95% CI, 0.71–0.93], *p* = 0.0003). The survival advantage was consistent in both age groups, with an encouraging difference in the over 65 group: HR for OS was 0.81 (95% CI, 0.60–1.08) in patients under 65 years and 0.82 (95% CI, 0.66–1.01) in those ≥ 65 years [40].

A pooled analysis using data from the studies KEYNOTE-010, KEYNOTE-024, and KEYNOTE-042 explored the efficacy and safety of Pembrolizumab compared to standard chemotherapy in the elderly population (aged ≥ 75 years) [41]. According to study protocols, all patients had a PD-L1 TPS of at least 1%; all patients were PS ECOG of 0–1, without symptomatic brain metastases or leptomeningeal disease and without other clinically relevant conditions. A total of 264 patients were aged ≥75 years, and 2348 were <75 years (patients aged 75 years or older were only the 11% of the total population). Among patients aged ≥75 years, 50% (n: 132) had PD-L1 TPS ≥ 50% and 65.2% had nonsquamous histology; the median age was 77 years (range, 75–90). With a median follow-up time of 11.7 months (range, 0.3–36.0) in the elderly population and 12.9 months (range, 0.1–38.3) in younger patients, the median treatment duration was 5.6 months (0.03–34.8) for Pembrolizumab and 3.5 months (0.03–29.5) for chemotherapy among elders and 4.3 months (0.03–37.5) for Pembrolizumab and 3.5 months (0.03–37.0) for chemotherapy among youngers. Pembrolizumab improved OS, regardless of age and PD-L1 TPS. Among elderly patients, in the group with PD-L1 TPS ≥ 1%, mOS was 15.7 months with Pembrolizumab (95% CI, 10.7–20.2) and 11.7 months with chemotherapy (95% CI, 8.4–15.8), HR, 0.76 (95% CI, 0.56–1.02); in the group of PD-L1 TPS ≥ 50%, mOS was 23.1 months with Pembrolizumab (95% CI, 11.9-not reached) and 8.3 months with chemotherapy (95% CI, 7.0–11.1), HR 0.40 (95% CI, 0.25–0.64). Among younger patients with PD-L1 TPS ≥ 1%, mOS was 14.6 months with Pembrolizumab (95% CI, 13.1–16.6) and 11.1 months with chemotherapy (95% CI, 10.0–11.9), HR, 0.76 (95% CI, 0.69–0.84); in the group with PD-L1 TPS ≥ 50%, mOS was 19.2 months with Pembrolizumab (95% CI, 16.4–22.4) and 11.9 months with chemotherapy (95% CI, 10.1–13.1), HR, 0.67 (95% CI, 0.57–0.78). Across each individual study, the OS benefit of Pembrolizumab versus chemotherapy was shown in both elderly and younger patients, despite the smaller numbers of patients in some of the groups and the wide CI. Moreover, previously untreated patients with a PD-L1 TPS ≥ 50% from the KEYNOTE-024 and KEYNOTE-042 studies were considered. Among the 93 elderly patients, OS was improved adding Pembrolizumab: mOS 27.4 months with Pembrolizumab (CI 95%, 10.6-not reached) versus 7.7 month with chemotherapy (95% CI, 6.1–11.1), HR, 0.41 (95% CI, 0.23–0.73). Among younger patients, mOS was 20.0 months with Pembrolizumab (95% CI, 16.7–25.1) and 13.0 months with chemotherapy (95% CI, 11.3–15.4), HR, 0.71 (95% CI, 0.59–0.87). No new safety signals were reported. Among the 254 elderly patients (149 treated with Pembrolizumab and 105 with chemotherapy), the incidence of treatment-related AEs (TRAEs) was lower in the Pembrolizumab group compared with chemotherapy: 68.5% (102 patients) versus 94.3% (99 patients). Fatigue was the most common TRAE among elderly patients who received Pembrolizumab (17.4%), followed by decreased appetite and pruritus (12.8% each). Further, AEs of grade ≥ 3 were less common with Pembrolizumab compared with chemotherapy (24.2% versus 61.0%), as well as serious adverse events (SAEs) (16.1% versus 26.7%). Two treatment-related deaths occurred in 2 elderly patients in each treatment group (1.3% for Pembrolizumab versus 1.9% for chemotherapy). Discontinuation due to AEs occurred in 16 patients in each treatment group (10.7% for Pembrolizumab versus 15.2% for chemotherapy), 6 were due to pneumonitis and 2 to increased alanine aminotransferase. Immune-related AEs (irAEs) and infusion reactions of special interest were more frequent with Pembrolizumab with similar frequency in the two age groups. The most common irAEs were hypothyroidism (elderly, 8.7% versus younger, 10.4%), pneumonitis (7.4% versus 6.8%), and hyperthyroidism (5.4% versus 5.7%); G3–G4 irAEs and infusion reactions occurred in 14 elderly patients (9.4%) and 90 younger patients (6.8%) following treatment with Pembrolizumab. No G5 irAEs and infusion reactions in elders were registered. Interestingly, among previously untreated elderly patients whose disease progressed, about half of them (46.2%, 18/39) received subsequent therapy [41].

First-line Atezolizumab was compared to standard platinum-based chemotherapy in the randomized phase 3 trial IMpower110 in previously untreated patients with PD-L1 expression of at least 1% of the tumor cells (TC) or of the tumor-infiltrating immune cells (IC). Both squamous and non-squamous subtypes were included [60]. Median OS was significantly longer in the Atezolizumab group than in the chemotherapy group among patients with EGFR and ALK WT tumors and high PD-L1 expression (at least 50% of TC or at least 10% of tumor-infiltrating IC). Among patients aged 65–74 years, the HR was 0.63 (95% CI 0.34–1.19); patients older than 74 were only 23 (11.2%), and HR in this subgroup was 0.79 (95% CI 0.18–3.56) [60]. At the updated OS analysis, the advantage of Atezolizumab was confirmed in the high PD-L1 expression WT group, continued OS improvement: stratified HR 0.76, 95% CI 0.54–1.09 [42]. This advantage was confirmed among patients aged 65–74 years (median OS 20.2 versus 10.4 months; HR 0.78, 95% CI 0.45–1.36), but not in those aged over 74 (25.6 months versus NE; HR 1.03, 95% CI 0.31–3.48) [42]. Atezolizumab showed a more favorable safety profile than chemotherapy [42,60], and no safety signals in older patients were noted.

EMPOWER-Lung 1 is a phase 3 trial that compared the anti-PD1 Cemiplimab versus standard platinum-doublet chemotherapy in patients with advanced NSCLC. Crossover from chemotherapy to Cemiplimab was allowed at disease progression. Primary endpoints were OS and PFS assessed in the intention-to-treat population (ITT) and in a prespecified PD-L1 of at least 50% population (22C3 assay). In the PD-L1 ≥ 50% population (563 patients), Cemiplimab resulted in better survival: HR 0.57 (95% CI 0.42–0.77), *p* = 0.0002. Further, median PFS was better with Cemiplimab: HR 0.54 (95% CI 0.43–0.68, *p* < 0.0001. Significant improvements in overall survival and progression-free survival were observed in the ITT population, despite a high crossover rate (74%). In the subgroup analysis, patients were divided into two age groups: under 65 and equal to or above 65 years. Cemiplimab resulted in better OS in both age groups: HR 0.66 (95% CI 0.44–1) in the <65 years group; HR 0.48 (95% CI 0.30–0.76) in the ≥65 years group. The safety profile of Cemiplimab was consistent with the known profile of other PD-1 or PD-L1 inhibitors, and it was better than that of chemotherapy, as well as the patient-reported health-related QoL. No safety evaluation was reported by age groups [43]. At the 3-year follow-up, Cemiplimab demonstrated further in OS and in patients with advanced NSCLC with PD-L1 ≥50%, despite a crossover rate of 75%: mOS 23.4 months (95% CI, 19.4–27.4) versus 13.7 (95% CI, 11.2–16.2), HR, 0.63 (95% CI, 0.52–0.77); *p* = 0.0001 [61]. No age distinction has been reported for this updated analysis.

The IPSOS study is a phase III trial of first-line Atezolizumab versus single-agent chemotherapy in patients not eligible for a platinum-containing regimen [44]. Patients with NSCLC without driver mutations (EGFR or ALK), with any PD-L1 TC or IC level and ineligible to platinum-based chemotherapy due to poor PS (ECOG ≥ 2) or to age (≥70 years), were randomized 2:1 to Atezolizumab every 3 weeks or single-agent chemotherapy Vinorelbine (oral or intravenous) or Gemcitabine. ECOG PS 0 or 1 was permitted if ≥70 years of age with substantial comorbidities or other contraindications to platinum chemotherapy. In total, 453 patients were randomized; median age was 75 years (range, 33–94), 31% were ≥80 years and 83% had ECOG PS ≥ 2. Atezolizumab significantly improved OS (stratified HR, 0.78; 95% CI: 0.63, 0.97; *p* = 0.028), with 24.3% of patients alive at 2 years, versus 12.4% of patients treated with Vinorelbine. The survival benefit for Atezolizumab was observed in the group of patients under the age of 70 years (HR 0.75, 95% CI 0.49–1.14), in the group of patients aged 71 to 79 years (HR 0.68, 95% CI 0.49–0.94), but not in patients aged 80 or over (HR 0.97, 95% CI 0.66–1.44). This benefit was observed regardless of PD-L1 TPS. Further, ORR was better with Atezolizumab: 16.9% (95% CI 12.8–21.6) versus 7.9% (95% CI 4.2–13.5) and median DOR was 14.0 (95% CI 8.1–20.3) versus 7.8 (95% CI 4.8–9.7) months. Grade 3/4 TRAEs occurred in 16.3% and 33.3% of patients and G 5 TRAEs in 1.0% and 2.7% of pts in the Atezoliumab and chemotherapy arms, respectively. Moreover, Atezolizumab improved the time to confirmed deterioration (TTCD) of chest pain vs. chemotherapy (HR, 0.51; 95% CI: 0.27, 0.97); meaningful improvements from baseline were also seen for appetite loss and cough [44].

## 6. First-Line ICIs-Chemotherapy Combinations

Chemotherapy has been added to immunotherapy in order to increase the efficacy of single agent ICI.

Pembrolizumab demonstrated to prolong survival when added to chemotherapy in two phase 3 trials. In the KEYNOTE-189 patients with nonsquamous NSCLC without sensitizing *EGFR* or *ALK* mutations received 4 courses of platinum-Pemetrexed plus Pembrolizumab followed by maintenance with Pemetrexed plus Pembrolizumab in responders and in those with stable disease (up to 35 cycles, ∼2 years). Comparator was standard chemotherapy with Pemetrexed maintenance [62]. In the KEYNOTE-407 patients with SCC were treated with Carboplatin-Nabpaclitaxel or Paclitaxel plus Pembrolizumab for 4 courses followed by Pembrolizumab monotherapy in responders and in those with stable disease (up to 35 cycles, ∼2 years) [46]. In the KEYNOTE-189 Pembrolizumab favored both OS and PFS in both age groups: HR for OS 0.49 (95% CI, 0.37–0.65) in the younger group (<65 years) and 0.72 (95% CI, 0.54–0.97) in the elder group (≥65 years); HR for PFS 0.42 (95% CI, 0.32–0.55) in the younger group and 0.63 (95% CI, 0.48–0.83) in the elder group [45]. Further, in the KEYNOTE-407 Pembrolizumab favored both OS and PFS in both age groups: HR for OS 0.52 (95% CI, 0.34–0.80) in the younger group (<65 years) and 0.74 (95% CI, 0.51–1.07) in the elder group (≥65 years); HR for PFS 0.50 (95% CI, 0.37–0.69) in the younger group and 0.63 (95% CI, 0.47–0.84) in the elder group [46]. The addition of Pembrolizumab did not increased the frequency of AEs that are commonly associated with chemotherapy and the incidence of most irAEs was not higher with than that previously observed with Pembrolizumab monotherapy with the exception of nephritis and acute kidney injury, both associated also to Pemetrexed and platinum-based drugs [62]. No safety data were reported among the over 65 years old population in both studies [46,62].

EMPOWER-Lung 3 is a placebo-controlled, phase 3 study, which examined the efficacy and safety of Cemiplimab plus platinum-doublet chemotherapy in previously untreated NSCLC patients, irrespective of PD-L1 expression or histology [47]. Patients were randomized (2:1) to receive Cemiplimab or placebo for up to 108 weeks in combination with four cycles of platinum-doublet chemotherapy (instigator’s choice) with pemetrexed maintenance in those with nonsquamous histology. The addition of Cemiplimab to standard chemotherapy demonstrated to improve survival compared with chemotherapy alone. Considering the analysis by age subgroups, the survival advantage was less evident among patients with age ≥ 65 years: OS HR 0.57 (95% CI, 0.40–0.81) in patients aged <65 years, OS HR 0.88 (95% CI, 0.56–1.37) in patients aged ≥65 years. Change from baseline and time to definitive clinically meaningful deterioration in patient-reported pain symptoms were superior with Cemiplimab plus chemotherapy associated to maintenance of global health status and QoL. Safety profile was consistent with what previously reported for Cemiplimab monotherapy and for platinum-based chemotherapy [47].

In the IMpower131 phase 3 trial the addition of Atezoluzumab to Carboplatin-Nab-Paclitaxel (A + CnP) demonstrated to improve PFS (primary endpoint), but not OS, in patients with advanced squamous histology [48,63]. Elderly patients were under-represented since only 39 patients aged 74–85 (11.4%) were enrolled in the A + CnP arm. HR for PFS were: 0.77 (95% CI, 0.61–0.99) among patients aged <65 years (mPFS 6 versus 5.6 months); 0.66 (95% CI, 0.51–0.87) among patients aged 65–74 years (mPFS 6 versus 5.6 months); 0.51 (95% CI, 0.3–0.84) among patients aged 75–84 years (mPFS 7 versus 5.6 months). HR for OS were: 0.89 (95% CI, 0.68–1.15) among patients aged <65 years (mOS 14 versus 12.7 months); 0.84 (95% CI, 0.63–1.13) among patients aged 65–74 years (mOS 14.8 versus 14.4 months); 0.74 (95% CI, 0.45–1.23) among patients aged 75–84 years (mOS 13.9–11.1 months). The safety profile of the combination was consistent with the known safety profile of each individual medicine, and no new safety signals were identified [48].

The IMpower 150 phase 3 trial compared Carboplatin, Paclitaxel, Bevacizumab and Atezolizumab (ABCP) with Carboplatin, Paclitaxel, Bevacizumab in advanced stage nonsquamous NSCLC patients (including those with EGFR and alterations with disease progression or unacceptable toxicity from at least one approved tyrosine kinase inhibitor); after 4 cycles of induction, Atezolizumab and Bevacizumab or Bevacizumab alone were administered until disease progression or unmanageable toxicity [64]. Median age was 63, 149 patients (37.2%) were aged 65–74, 33 (8.2%) were aged 75–84, and 3 (0.8%) were older than 85. In the ITT wild type population HR for OS were: 0.83 (95% CI, 0.65–1.04) among patients aged <65 years (mOS 18.9 versus 14.3 months); 0.72 (95% CI, 0.54–0.97) among patients aged 65–74 years (mOS 22.9 versus 15.8 months); 0.97 (95% CI, 0.58–1.62) among patients aged 75–84 years (mOS 16.7–14.1 months) [49]. Further, PFS, which was the primary endpoint favored ABCP combination. HR for PFS were: 0.65 among patients aged < 65 years (mPFS 8 versus 6.8 months); 0.52 among patients aged 65–74 years (mPFS 9.7 versus 6.9 months); 0.78 among patients aged 75–84 years (mPFS 9.7 versus 6.8 months). The safety profile of ABCP was consistent with the ones of each drug, including the rate of hemorrhagic events caused by Bevacizumab; no new safety signals were observed with the combination and the rate of irAEs was as expected [64].

## 7. First-Line ICI-ICI Combinations

Dual immune checkpoint inhibition represents an intriguing strategy in the treatment of NSCLC. The combination of anti-CTLA4 with anti-PD1/PDL1 antibodies enhances antitumor activity early at sites of T-cell activation and in the tumor microenvironment through effector T-cell responses; moreover, it improved the ratios of effector to regulatory T cells and myeloid-derived suppressor cells in preclinical melanoma model, suggesting an overall enhancement of antitumor immunity [65,66,67]. Results from the most relevant clinical trials in this setting are reported below and summarized in Table 1.

In the open-label phase 3 trial CheckMate, 227 previously untreated patients with stage IV or recurrent NSCLC and a PD-L1 TPS of at least 1% were randomly assigned to receive Nivolumab plus Ipilimumab or Nivolumab alone or chemotherapy; those with a PD-L1 expression < 1% were randomly assigned to Nivolumab plus Ipilimumab or Nivolumab plus chemotherapy or chemotherapy alone. First-line treatment with Nivolumab plus Ipilimumab resulted in a longer OS than did chemotherapy, independently of the PD-L1 TPS [68]. In patients with a PD-L1 TPS ≥ 1%, mOS was 17.1 months (95% CI, 15.0–20.1) with ICIs combination versus 14.9 months (95% CI, 12.7–16.7) with chemotherapy, HR 0.79 (0.75–0.96), *p* = 0.007 [68]. The same benefit was also observed in patients with a PD-L1 expression level < 1%: mOS 17.2 months (95% CI, 12.8 to 22.0) versus 12.2 months (95% CI, 9.2–14.3), HR 0.62 (95% CI, 0.48–0.78) [68]. The percentage of grade 3 or 4 TRAEs in the overall population was similar in both groups: 32.8% with Nivolumab plus Ipilimumab and 36.0% with chemotherapy. Treatment-related SAEs of any grade were more common with ICIs combination (24.5% versus 13.9%), as well as TRAEs leading to discontinuation (18.1% versus 9.1%). The most common immune-related TRAEs were skin reactions (34%) and endocrine events (23.8%). Treatment-related deaths occurred in 8 patients who received Nivolumab plus Ipilimumab and in 6 patients treated with chemotherapy [68]. The median age in the overall population was 64 years (range 26–87). Patients aged ≥75 years accounted only for 9% of the total. In the Nivolumab plus Ipilimumab group, patients aged 75 and over were 58 (9.9%), those aged 65–75 were 219 (37.6%), and those under the age of 65 were 306 (52.5%); patients aged 65 and over were globally 47.5%. Among patients under 65 years of age the OS benefit of nivolumab plus ipilimumab was evident regardless of PD-L1 expression: HR 0.70 in PD-L1 ≥ 1% and 0.69 in PD-L1 < 1%. This benefit seems to get lost advancing with age: in the PD-L1 positive group HRs were 0.91 among patients aged 65–74 and 0.92 among patients aged 75 or more; in the PD-L1 negative group HRs were 0.49 among patients aged 65–74 and 0.75 among patients aged 75 or more. More patients aged 65 and over were enrolled in the PD-L1 positive group (387 versus 168) [68]. At longer follow-up, Ipilimumab plus Nivolumab continued to show durable clinical benefit with increased 5-year survivorship versus chemotherapy regardless of PD-L1 TPS: after a minimum follow-up of 61.3 months, 5-year OS rates were 24% versus 14% for Nivolumab plus Ipilimumab versus chemotherapy (PD-L1 ≥ 1%) and 19% versus 7% (PD-L1 < 1%); among those alive at 5 years, 66% (PD-L1 ≥ 1%) and 64% (PD-L1 < 1%) were off Nivolumab plus Ipilimumab without initiating subsequent systemic anticancer treatment by the 5-year time point, and survival benefit continued also after ICIs discontinuation because of TRAEs [50]. Moreover, QoL in 5-year survivors treated with Nivolumab plus Ipilimumab was similar to that in the general US population [50].

A pooled analysis evaluated the safety of first-line Nivolumab plus Ipilimumab (Nivolumab 3 mg/kg or 240 mg every 2 weeks; Ipilimumab 1 mg/kg every 6 weeks) in a large population from three first-line studies [69]: CheckMate 227 part 1 [68], CheckMate 817 cohort A [70,71,72], and CheckMate 568 part 1 [73]. The pooled population accounted for 1255 patients. The median age was 65 years (range 26–91); patients aged 75 years or older were 174 (14%). Any-grade TRAEs occurred in 78% of the patients and G3-G4 TRAEs in 34%; discontinuation of any regimen component owing to TRAEs in 21%. Compared to the general population, more patients aged ≥75 years had an ECOG PS of 1 (72% vs. 61%); they received a median of 8.0 doses of Nivolumab (range 1–55) and 3.0 doses of Ipilimumab (range 1–19), with a median duration of treatment of 3.9 months (range 0–25.6). Similar to the overall pooled population, the main reasons for discontinuation were disease progression (38%), drug toxicity (29%), and completed treatment (14%). Safety in patients aged ≥75 years was similar to the overall population. TRAEs of any grade occurred in 78% in both groups (elders and overall population). G3 and G4 TRAEs were more frequent (44% versus 34%), and TRAEs leading to discontinuation occurred in 29% of patients (versus 21%). OS in patients aged ≥75 years who discontinued treatment due to TRAEs was consistent with the overall population. Serious TRAEs were also numerically more frequent among elders (29% versus 23%; G3/G4 24% versus 18%). Treatment-related deaths occurred in two (1%) patients (1 myocarditis and 1 autoimmune esophagitis), and it was consistent with the overall population. Further, the incidence of any grade irAEs was comparable with the overall population and with patients under 75 years, occurring mainly within the first 6 months of treatment (as in the pooled population), with the exception of diabetes mellitus, which had a median time to onset of 9.4 months (range: 0.7–18.2). Similar to the pooled population, the irAE with the shortest median time to onset was hypersensitivity (0.5 months); other early irAEs were hyperthyroidism, nephritis/renal dysfunction, and rash (within the first 2 months of treatment). As with the overall population, most nonendocrine irAEs resolved (≥76%); adrenal insufficiency and hyperthyroidism were the only endocrine irAEs with most events resolved (75% and 57%, respectively). Corticosteroid use pattern to manage irAEs was similar among patients aged 75 years or older and the general population. Only one elder patient was treated with Infliximab for diarrhea/colitis, numerically lower when compared to the 4 patients treated with immunosuppressive treatment other than corticosteroids in the overall population. Globally, these data give evidence of a manageable safety profile of the ICIs combination Nivolumab plus Ipilimumab in this elder subgroup, without affecting clinical activity [69].

CheckMate 9LA is a phase 3 randomized open label trial that evaluated Nivolumab (360 mg intravenously every 3 weeks) plus Ipilimumab (1 mg/kg intravenously every 6 weeks) combined with histology-based, platinum doublet chemotherapy (every 3 weeks for two cycles), versus chemotherapy alone (every 3 weeks for four cycles) [74]. The trial met its primary endpoint showing a benefit in OS for the experimental group at pre-planned interim analysis after a median follow-up 9.7 months: HR 0.69 (96.71% CI, 0.55–0.87), *p* = 0.00065; it was confirmed after further 3.5 months: mOS 15.6 (95% CI 13.9–20.0) versus 10.9 months (95% CI 9.5–12.6), HR 0.66 (95% CI 0.55–0.80) [74]. Further, with an extended median follow-up of 30.7 months survival continued to favor experimental arm: mOS 15.8 versus 11.0 months, HR 0.72 (95% CI 0.61–0.86); 2-year OS rate was 38% versus 26% [51]. Two-year PFS rate was 20% versus 8%. ORR was 38% versus 25%, respectively; 34% versus 12% of all responses were ongoing at 2 years [51]. These results were independent from PD-L1 TPS [51]. Treatment was overall well tolerated: TRAEs of any grade occurred in 92% versus 88% of patients; grade 3/4 TRAEs were reported in 48% and 38%, respectively. The majority of grade 3/4 TRAEs in the experimental arm occurred during the first two cycles, corresponding to the duration of the limited course of platinum-doublet chemotherapy. TRAEs of any grade leading to treatment discontinuation were reported in 17% versus 6%. Grade 5 AEs were 2% in both arms (8 versus 6 patients). The most common irAEs of any grade were rash (17%), hypothyroidism/thyroiditis (16%), and hyperthyroidism (8%); the most common grade 3/4 irAEs were hepatitis, rash, and colitis (each 4%) [51,74]. Those who discontinued in the experimental arm due to TRAEs continued to benefit from the combination of ICIs [51]. Most enrolled patients were under the age of 65: 354 compared to 295 of those aged 65–75 and only 70 patients were older than 75 years. The survival benefit is maintained in the first two groups while it is lost for patients older than 75 years: mOS 8.5 (95% CI 5.6–13.5) versus 11.5 months (95% CI 5.8–15.2), unstratified HR 1.04 1.04 (95% CI 0.63–1.72) [51].

## 8. Observational and Real-World Setting Studies in Advanced Stage Disease

Several real-world (RW) retrospective and observational studies evaluated the activity of ICIs also in the elderly population.

A monocentric retrospective study evaluated the activity of PD1/PD-L1 inhibitors (any line of treatment) in 245 patients; 26.1% were <60 years, 31.4% were 60–69 years, 31.0% were 70–79 years, and 11.4% were ≥80 years. Charlson comorbidity index (CCI) scores were higher among older patients (mean CCI scores: age <60 years, 6.6; 60–69 years, 6.7; 70–79 years, 7.0; and ≥80 years, 6.9 [*p* = 0.011]). The median PFS times were: 1.81 months among patients under 60 years of age; 2.53 months among those aged 60–69 years; 3.75 months in the age group of 70–79 years; 1.64 months in patients aged 80 years or older (log-rank *p* value = 0.055). Multivariable Cox regression demonstrated that patients aged 70–79 years had a significantly lower hazard for disease progression or death than younger patients (HR 0.60, *p* = 0.015). Survival was similar among patients under 60 years of age, those with age of 60–69 years, and those with age 70–79 years (mOS 13.01, 14.56, and 12.92 months, respectively). Patients aged ≥80 years (only 28) had shorter mOS (3.62 months). Multivariable Cox regression demonstrated that patients aged ≥80 years had a significantly higher hazard for death compared with younger patients (HR 2.74, *p* = 0.002). No significant difference in rate of irAEs was found by age, as well as rates of steroid use and hospitalizations [75].

The efficacy and safety of Nivolumab monotherapy in patients with squamous NSCLC treated in the Nivolumab Expanded Access Programme (EAP) in Italy has been reported elsewhere; the study enrolled 371 patients evaluated in a real-world (RW) setting [76]. A subsequent analysis evaluated the efficacy and safety in elderly patients. In total, 126 patients (34%) were aged <65, 175 (47%) were aged 65–<75 and 70 (19%) were aged ≥75 years. The efficacy was similar across age groups. ORR was respectively of 18%, 18% and 19%, similar to the overall population (18%); DCR was 49%, 47% and 43% (47% in the overall population). Survival was lower in patients aged ≥75 years, with mOS of 5.8 months; it was 8.6 months in patients aged <65 years, 8 months in those aged 65–<75 years and 7.9 months in the overall population. Grade 3–4 TRAEs frequency was low: respectively 3%, 9%, 3% in the three age groups and 6% in the overall population. Further, discontinuation rates due to TRAEs were low [77].

A similar study reported the data of 1588 Italian patients with pretreated advanced non-squamous NSCLC, who received Nivolumab in the context of the EAP [78]. In the global population, ORR rate and DCR were 18% and 44% respectively; mOS was 11.3 months (95% CI, 10.2–12.4). Elderly patients (≥70 years n: 522; ≥75 years n: 232) achieved outcomes similar to the overall study population: among the 456 patients aged ≥70 years evaluable for response, ORR and DCR were 21% and 48% respectively; mOS was 11.5 months (95% CI: 10.0–13.0), and OS rate at 1 year was 48%. Among the 212 patients aged ≥75 years evaluable for response, ORR and DCR were 25% and 53% respectively; median OS was 12.0 months (95% CI: 9.2–14.8), and OS rate at 1 year was 50% [78].

Similarly, age was not a predictor of survival among 902 French patients treated with Nivolumab as second or later line in the French EAP, from French Cooperative Thoracic Intergroup (IFCT) 1502-CLINIVO [79].

A Japanese cohort of 45 NSCLC patients aged 80 years and over treated with Pembrolizumab, Nivolumab, or Atezolizumab in first or subsequent lines of therapy was evaluated retrospectively [80]. Four patients with EGFR activating mutation were included. Median age was 85 years (range 80–94). Most patients received Nivolumab as second or subsequent line (47%), and only the 15.6% of patients were treated in first line, all of them with Pembrolizumab. Most had PS ECOG ≥ 2 (36%). PFS was 3.4 months (0.2–17.8); PR were the 22.2%, SD were the 37.8% with a disease control rate of 60%. Pembrolizumab resulted in the best response rate (PR 35.3%), with a DCR of 76.5%; the majority of patients who received Pembrolizumab had a PD-L1 TPS ≥ 50% (70.6%). irAEs of grade ≥ 2 were 7 (15.5%). One G5 pneumonia was registered with Nivolumab. Other 3 G3 pneumonia occurred with Pembrolizumab. Atezolizumab showed the best safety profile. Taken together, with the limitation of the small sample size, these results confirm the possibility of treating a very elderly population with ICIs [80].

A retrospective study on 125 consecutive patients treated with Pembrolizumab or Nivolumab (any line of treatment) in Japan evaluated the effectiveness and safety in older patients or those with poor PS [81]. The median age was 60 years (range, 31–85). The elderly group (≥75 years) numbered only 15 patients (12%). No significant differences in the ORR or DCR between the two age groups were found (*p* 0.232 and *p* 0.418 respectively). Further, survival did not differ between older and younger patients. HR for PFS and OS were 1.15 (95% CI, 0.63–2.12; *p*: 0.647) and 1.06 (95% CI, 0.9; *p* 0.858), respectively. In multivariate analysis, poor PS and later line of therapy (3rd) were independent factors that predicted poor survival. Moreover, the rate of AEs and AEs grade ≥ 3 did not differ between the two age groups: AEs 73.3% versus 65.5% (*p* 0.77), AEs G ≥ 3 33.3% versus 12.7% (*p* 0.526). Severe pneumonitis was more frequent in the older group, as it was reported in 3 patients, although this difference was not statistically significant (20.0%, *p* 0.0546) [81].

Another Japanese retrospective study analyzed the clinical factors associated with shorter durable responses to first-line Pembrolizumab in 88 patients with PD-L1 TPS ≥ 50% and who experienced initial response [82]. Older age (≥75 years) was associated with shorter PFS (mPFS 10.0 versus 20.6 months, HR 1.96; 95% CI, 1.11–3.47; *p* 0.020). However, only 26 elderly patients were included, representing 29.5% of the entire population [82].

Grosjean and coll. reported the outcomes of a retrospective cohort of patients from the Alberta Immunotherapy Database in a RW population with NSCLC and PD-L1 TPS ≥ 50% treated with first-line Pembrolizumab [83]. Among 327 patients, 169 (51.7%) were older than 70 years. Compared to younger patients, older adults were more likely to have a lower stage of disease at the time of their initial diagnosis (*p*: 0.03); no other significant differences were found. After a median follow-up time of 19.2 months (2.7–41.7), mOS was 11.2 months (95% CI, 8.8–15.3), median TTF was 3.1 months (95% CI, 3.1–4.9), and ORR was 32.0%. There was no difference among the two age groups: mOS was 11.3 months (95% CI, 8.9–16.2) in older patients versus 11.2 (95% CI, 6.7–22.2) months in younger adults (*p* = 0.91); median TTF was 4.1 months (95% CI 2.8–6.0) in elders versus 3.46 (95% CI, 2.7–5.0) months in youngers (*p* = 0.98); ORR was 30.8% in older adults versus 33.3% (*p* = 0.67). Further, the exploratory analysis of the landmark survival times did not show differences. More patients in the younger group started a subsequent therapy at progression (24.1% versus 13.7%, *p* = 0.02). No differences were also found in the frequencies of significant irAEs: 26.6% versus 26.6% (*p* = 0.99). The most common irAEs were pneumonitis, colitis, and arthritis. Moreover, the rates of hospitalizations from any cause were similar: 44.9% in youngers versus 46.7% in older adults (*p* = 0.74). A sensitivity analysis considering patients aged ≥75 years did not find any significant differences in the clinical or safety outcomes, which were similar to the primary analysis. Age was not a prognostic factor of survival neither in univariate nor in multivariate analysis. This report confirms the efficacy and safety of first-line Pembrolizumab also in patients over the age of 70 [83].

A large Italian multicenter retrospective study including 1026 consecutive patients, evaluated the clinicopathologic correlates of Pembrolizumab effectiveness in previously untreated NSCLC patients with a PD-L1 expression ≥ 50% [84]. In the univariate analyses age (≥70 versus <70 years old) was not related to ORR (45.3% versus 43.6%, *p* = 0.6023), PFS (HR 1.01, *p* = 0.9983) or OS (HR 1.04, *p* = 0.6923) [84]. Safety was not evaluated in this study [84].

Another large analysis from the SEER-Medicare database identified 1256 patients aged ≥65 years who were diagnosed with pathologically confirmed stage I-IV NSCLC treated with Nivolumab or Pembrolizumab [85]. The median age at the time of ICIs initiation was 75.3 years; 8.4% of patients were aged ≥85 years. Most had nonsquamous histology (63.14% nonsquamous, 29.62% squamous). Five hundred and thirty-five patients (42.60%) had stage IV disease. Half of the patients (49.7%) initiated ICIs after platinum doublets and 8.1% had received no systemic therapy prior to ICI initiation. The mOS from the time of ICIs initiation was 9.3 months (95% CI, 8.5–10.5 months), the 1-year survival probability was 43.0% (95% CI, 40.2–45.7%). Factors associated with increased risk of death in multivariable survival analyses were: multiple comorbid conditions, squamous histology, a history of non-platinum doublet therapy, recent radiotherapy, and a shorter time from the initial diagnosis to treatment initiation [85].

Another large retrospective study was the one from the Dutch immunotherapy registry for patients with lung cancer [86]. A total of 2302 patients with advanced NSCLC who received with ICIs were analyzed. Median age was 63 and those aged ≥75 years were 242 (9.0%). Survival was not significantly different for patients aged >75 years: mOS 12.3 months (95% CI, 11.3–13.3) in the group aged 28–75 years (HR 1), 13.7 months (95% CI, 12.3–19.9) in the group aged >75–88 years (HR 0.84; 95% CI, 0.66–1.08). Factors associated to worse OS were PS ECOG ≥5, non-smoking history, palliative radiotherapy before immunotherapy [86].

Takamori S and coll. reported the activity of ICIs obtained from retrospective data of the National Cancer Database in US. A total of 86,173 patients with stage IV NSCLC were selected for the analysis. The 14.5% of patients aged <75 years (8968 of 62,037) and the 9.3% patients aged ≥75 years (2241 of 24,136) received ICIs. Survival was significantly higher among patients who received ICIs in both age groups: mOS 14.5 versus 7.8 months (HR 0.67, 95% CI 0.65–0.68; *p*  <  0.0001) among patients aged <75 years; mOS 11.9 versus 5.4 months (HR 0.61, 95% CI 0.58–0.64; *p*  <  0.0001) among patients aged ≥75 years. In the older age group, multivariate analysis of OS showed that male sex, white race, nonacademic institution, Charlson-Deyo score  ≥  2, non-adenocarcinoma NOS histology, nodal status  ≥  N1, brain metastasis, bone metastasis, liver metastasis, no surgery of the primary lesion, chemotherapy, and no ICI therapy were independent predictors of shorter OS [87].

Clinical outcomes and irAE patterns were evaluated in a multicenter international retrospective study on 928 geriatric patients with different tumors treated with single-agent ICIs [88]. NSCLC patients were 345 (37.2%), all treated with anti-PD1/PD-L1 agents. In the NSCLC cohort median age at ICI initiation was 82.9 years (range 78.4–95.0), with 252 patients (73.0 %) younger than 85, 83 (24.1%) aged 85–89 years, and 10 (2.9%) aged 90 years or older. Documented response data were available for 276 NSCLC patients; ORR was 32.2% (3.6% CR, 28.6% PR); ORR in patients with NSCLC younger than 85 and 85 years or older was 34.5% vs. 25.7% respectively (*p*  =  0.18). In the overall NSCLC cohort, mPFS was 6.7 months (95% CI, 5.2–8.6 months) and mOS was 10.9 months (95% CI, 8.6–13.1 months). In those younger than 85 years and aged 85 years or older, the median PFS was 8.0 (95% CI, 5.6–9.5) and 5.0 (95% CI, 4.0–8.4) months, respectively (*p*  =  0.40). The median OS was 11.8 (95% CI, 9.3–15.3) months vs. 7.5 (95% CI, 5.0–11.5), months, respectively (*p*  =  0.047). In the overall population (928 patients) similar rates of any-grade irAEs were found among age groups, as well as G3/G4 irAEs. Patients aged 90 years or older were more likely to discontinue treatment due to irAEs than those younger than 90 years (30.9% vs. 15.1%, *p*  =  0.008) [88].

The efficacy and safety of Anti-PD-1 therapy was retrospectively evaluated in 86 patients aged ≥75 years treated in 7 Italian institutions. The median age was 78.5 years (range, 75–86 years); the majority of them (80.2%) had a PS ECOG 0 or 1; the mean number of comorbidities was 2.9 (range, 0–8); the number of mean drugs taken at baseline before staring immunotherapy was 3.66 (range, 0–11); the 79.1% of patients were pre-treated. The 73% of patients were treated with Nivolumab and 27% with Pembrolizumab. The overall disease control rate was 65.1%. The median OS was 10.1 months (range, 1.7–34.8; SD, 8 months). No difference in survival was found between patients younger and older than 80 years. The rate of irAEs was 17.4% (fatigue and rush were the most frequent followed by hepatitis and pruritus). The only grade ¾ toxicities were diarrhea and neutropenia (both occurring in only 1 patient). Patients with PS 2 experienced the same rate of irAE (17%); grade ¾ toxicities occurred in 5% of patients (one G4 neutropenia). This study strengthened the feasibility of immunotherapy in old and very old patients in common clinical practice, even if it was a good clinically selected population [89].

In an Asian retrospective study on 141 patients (median age 63, range 39–85), age was not a significant predictor of irAEs from anti-PD-1 or anti-PD-L1 treatment, both in univariate and multivariate regression analysis for any grade IrAE (age ≥70 versus <70). Incidence ratio (IR) was 1.456 (95% CI, 1.085–1.954; *p* = 0.012) in the univariate analysis and 1.139 (95% cI, 0.805–1.612; *p* = 0.462) in the multivariate analysis. Age was not even associated with survival [90].

An interesting French study evaluated prospectively the frequency of irAEs in patients affected by solid tumors (melanoma, NSCLC and urothelial carcinoma) and treated with ICIs in a real-life setting, distinguishing young patients (aged < 70 years) and old patients (aged ≥70 years) [91]. Data were collected between June 2014 and October 2017 from the REISAMIC registry (Registre des Effets Indesirables Severes des Anticorps Monoclonaux Immunomodulateurs en Cancerologie) which prospectively collect clinical and biological data from patients treated with ICIs and targeted antibodies who experienced grade ≥ 2 irAEs. Patients included were 603: 191 old patients (32%) and 412 young patients (68%). The median ages were 77 (range 70–93) and 59 (range 17–69) years respectively. The incidence of irAEs grade ≥ 2 was higher between the elders (33% versus 25%, *p* = 0.035). The statistical significance was lost when stratifying irAEs in accordance with their severity but there was a trend for a higher incidence of irAEs of lower grades between elders (*p* = 0.08 for G2 and *p* 0.13 for G3–4). Interestingly, there was a difference in organs affected: skin (49%), endocrine (14%) and liver (10%) in the old group; skin rash (28%), endocrine (25%) and digestive (15%) in the young group. The incidence of skin toxicities was much higher in the old group (*p* = 0.007), while the endocrine toxicities were more frequent in the young group (*p* = 0.044). Elders experienced a higher incidence of multiple irAEs of grade ≥ 2 per individual: 20/63 (32%) versus 18/103 (18%), *p* = 0.037. Discontinuation rate was 32% in the old group and 25% in the young group (the difference is not statistically significant). Discontinuation rate due irAEs was slightly higher in the old group (14% versus 7%, *p* = 0.2). G5 events were 1 in the OG and 3 in the YG (0.5% and 0.7%, respectively, not statistically significant). The median time to toxicity was not statistically different between the two groups: 6 weeks in the old group versus 10 weeks in the young group (*p* = 0.13). No differences were found in clinical outcome in the 2 cohorts: median PFS was 8.0 months in the old group (95% CI 5.9–15.7) and 6.0 months in young group (95% CI: 5.5–6.9), *p* = 0.126. Further, mOS was not statistically different: 12.0 months in the old group (95% CI, 8.5–21.3) and 9.0 months in the young group (95% CI, 7.1–10.2), *p* = 0.07. Moreover, when considering NSCLC patients, survival did not differ [91].

The ELDERS study is a prospective observational study designed to assess the safety of ICIs in older cancer patients, explore predictive factors and the role of geriatric assessments [92]. Patients with advanced/metastatic NSCLC or malignant melanoma candidate to start ICIs monotherapy in any treatment line were recruited and divided (1:1) into 2 age cohorts: older (≥70 years) and younger (<70 years). The study-specific assessments were completed at baseline and every 3 months with an observation period of 12 months. Comorbidity and polypharmacy (≥5 concomitant medications) were assessed for all patients. The Cumulative Illness Rating Scale adapted for Geriatrics (CIRS-G) measured the comorbidities by organ system. Geriatric assessments were performed in the older cohort at baseline and repeated every 3 months and were based primarily on the G8 screening tool. A positive G8 screening (<15 points) triggered a set of holistic geriatric assessments (repeated at each subsequent review). Geriatric interventions were not planned. A total of 140 patients were enrolled; the median follow-up time was 6.3 months. Only 52 patients (37%) completed the planned 12 months on study. The 85% of patients who stopped the study earlier it was due to progressive disease (PD). The older cohort had a higher incidence of polypharmacy (*p* = 0.004) and comorbidity burden (*p* < 0.001). The incidence of G3–4 comorbidities was higher in the older cohort (77% versus 56%; *p* = 0.008). Geriatric assessments were completed in all older cohort; 35 patients (50%) had a positive G8 screening and 34 of them were identified at the baseline assessment; all repeated the geriatric assessments at each review. The patients with negative screening (fit subgroup) at baseline remained negative at the subsequent reviews. Those with a positive screening (frail subgroup) were overall older (*p* = 0.056), had a worse performance status (PS) (*p* < 0.001), a higher comorbidity burden (*p* = 0.001) and more polypharmacy (*p* = 0.001); there were no differences in cancer burden. The 60% within this frail subgroup (n: 21) would be classed as fit if based solely on the standard PS assessment of 0 or 1. Besides comorbidity and polypharmacy, the most commonly affected component was the capacity to perform activities of daily living (ADL): 66%; all of them had issues performing instrumental ADL, such as shopping and cooking, but 17% also reported limitations with basic activities, such as eating or going to the toilet. Considering those with at least two sets of holistic geriatric assessments completed (23/35), 19 (83%) were either stable or had an improvement in the affected component(s) where issues were identified. Geriatric interventions directed at affected components occurred outside of the study and their impact was not evaluable on this study. The incidence of irAEs were slightly higher in the older cohort but not significantly different. Any grade irAEs were 60% versus 51.4% (odds ratio 1.41, CI 95% 0.69–2.92; *p* = 0.395); grade 3–5 irAEs (primary endpoint) were: 18.6% versus 12.9% (odds ratio 1.55, CI 95% 0.61–3.89, *p* = 0.353). One G5 pneumonitis event occurred in the older cohort. No difference in the profile of irAEs was found and exposure to systemic steroids was slightly longer in older group but not significant: median 22 weeks (CI 95% 9.5–34.5) versus 8 weeks (CI 95% 5.3–10.7), *p* = 0.208. Further, the discontinuation rate, the incidence of non-irAEs and hospital admission rates were similar. In the multivariate analysis no patient-related factors or cancer burden were predictive for key safety outcome. Higher comorbidity score and polypharmacy were associated with an increased risk of death (*p* = 0.04 and *p* = 0.03, respectively). A positive G8 screening in the older cohort was a predictor for hospital admissions (*p* = 0.031) in multivariate analysis. Only 32% of admissions of patients in the frail group were treatment related; the remaining admissions were due to other non-irAEs (such as infections, thrombotic events, falls, pain), whereas for those in the fit subgroup, 58% of admissions were treatment related. A positive G8 screening was also associated with higher risk of death (*p* = 0.01) [92].

Retrospective RW studies have been reported also for combination regimens.

Fujimoto D and coll. investigated the effectiveness and safety of combination therapy of Pembrolizumab plus chemotherapy in patients with previously untreated nonsquamous NSCLC in a RW setting [93]. Patients enrolled were 299; median age was 68 years; 43 were aged ≥75 years (14.4%). In the elderly group most patients were male (65%) and former smokers (44%). After a median follow-up of 11.7 months, median PFS of those aged < 75 and ≥ 75 years were 8.5 (95% CI, 7.0–9.9) and 8.9 (95% CI, 6.7–10.5), respectively. OS was not reached in both groups. In the multivariate analysis, age (≥75 years versus <75 years) was not an independent factor associated to PFS. Severe aEs were more frequent between elders: 26% in the elderly group versus 19% in those aged < 75 years, although the difference was not significant (*p* = 0.312). The most frequent non-hematologic AE grade ≥ 3 was pneumonitis (5%). The rate of discontinuation of all treatment components due to aEs was significantly higher among the elderly: 40% versus 21% (*p* = 0.012). Dividing patients into age groups (<65, 65–74, ≥75), incidence of aEs and discontinuation rates increase with age. The main event leading to discontinuation was pneumonitis in the overall population. A total of 10 G5 events were recorded, two in the elder group (4 pneumonitis, 2 febrile neutropenia, 2 sepsis, 1 lung infection and 1 death not otherwise specified). Taken together these results, even if on a small number of elderly patients treated in real life, confirm the efficacy of the KEYNOTE-189 regimen also in an elderly population, but highlights the increased toxicity in this population [93].

Another retrospective monocentric observational study on 136 NSCLC Chinese patients aged ≥75 years has been recently published [94]. In total, 43 patients were treated with Pembrolizumab plus chemotherapy and 93 with monochemotherapy. Median age was 77.9 years in the first group (range 75–85) and 77.3 years in the second (range 75–88). More patients were PD-L1 positive (TPS ≥ 1%) in the combination group (46% versus 24%), while PD-L1 negative and PD-L1 unknown were more frequent in chemotherapy group (28% and 34% PD-L1 negative; 26% and 42% PD-L1 unknown). Objective response rate were significantly higher in the chemotherapy-Pembrolizumab group (53% versus 29% and 53%, *p* = 0.006). Further, PFS was significantly longer in the combination group (12.50 versus 5.30 months, *p* < 0.001) as well as OS (unreached versus 21.27 months, *p* = 0.037). PD-L1 positive patients, reached better PFS and OS with Pembrolizumab-chemotherapy (PFS 12.53 versus 4.17 months, *p* < 0.001; OS unreached versus 18.30 months, *p* = 0.048). In patients with PD-L1 negative PFS and OS were slightly better in the combination group but the difference was not significant (PFS: 8.43 versus 4.85 months, *p* = 0.062; OS unreached versus 16.13 months, *p* = 0.71). In patients with unknown TPS, PFS was significantly better with combination regimen, whereas OS did not (mPFS 13.73 versus 5.77 months, *p* = 0.005; mOS unreached versus 22.80 months, *p* = 0.17). In the subgroup analysis patients with poor PS (ECOG ≥2), female gender and squamous histology did not reach significance for better PFS. In the multivariate analysis older age (≥80 years) (*p* = 0.016, HR = 2.17, 95%CI 1.16–4.08) and higher TNM stage (*p* = 0.010, HR = 2.01, 95%CI 1.18–3.43) had a negative effect on OS. There was no difference in the incidence of AEs grade ≥ 3 between the two groups (67% and 61% respectively *p* = 0.49). The most common AEs in both groups were neutropenia, thrombocytopenia, and anemia. Pneumonia (9% versus 0%, *p* = 0.009) and dyspnea (7% versus 1%, *p* = 0.093) were more common in the combination group. In the Pembrolizumab group 26% of patients discontinued due to AEs (6 due to pneumonia of any grade; 1 each to thrombocytopenia, myocarditis, hypothyroidism, skin rash and anemia). No difference in G5 events were registered (5% versus 4%, *p* = 0.93). Taken together these results confirm the efficacy and safety of the combination regimens also in an elderly population (age ≥ 75), even if with a small sample size and without any specification of the chemotherapy regimen associated with Pembrolizumab [94].

## 9. Locally Advanced Disease

Concurrent chemo-radiotherapy (cCRT) represents the gold standard for the treatment of unresectable locally advanced NSCLC (LA-NSCLC) in patients with good performance status; it has been proven to be better than sequential CRT (sCRT) [95]. The advantage of cCRT was demonstrated also in patients older than 70 years [95]. No major advances have been made in this setting for many years, up to the demonstration of the effectiveness of combining ICIs with radiotherapy.

The PACIFIC study is a randomized double-blind phase III trial that compared consolidation therapy with the anti-PDL1 Durvalumab versus placebo in unresectable LA-NSCLC patients who did not progress after platinum-based cCRT [96]. Consolidation Durvalumab improved progression free survival (PFS) [96] and overall survival [97] with a manageable safety profile. This advantage was confirmed also in subsequent updates [97,98,99,100]. At last exploratory analysis, approximately after 5 years after the last patient was randomized, mPFS was 16.9 vs. 5.6 months (stratified HR 0.55; 95% CI, 0.45–0.68), with an estimated 5-year PFS rate of 33.1% versus 19% [100]; mOS was 47.5 vs. 29.1 months with a 28% of reduction in the risk of death (stratified HR 0.72; 95% CI, 0.59–0.89) and an estimated 5-year OS rate of 42.9% versus 33% [100]. Further, ORR was higher with Durvalumab versus placebo: 29.8% vs. 18.3%, reflecting also in longer duration of response; at 5 years 51.1% of patients were estimated to have an ongoing response with Durvalumab versus none with placebo [100]. The safety profile of consolidation Durvalumab after cCRT is manageable and consistent with what observed for other ICIs. Any grade AEs were found in the same proportion in both arms, as well as G3/G4 AEs (29.9% with Durvalumab and 26.1% with placebo); the most G3/G4 AE common was pneumonia (4.4% vs. 3.8%) [96]. Discontinuation due to AEs occurred in 15.4% of patients in the Durvalumab group and 9.8% of patients in the placebo group; the most frequent AEs leading to discontinuation of Durvalumab or placebo were pneumonitis, radiation pneumonitis and pneumonia [96]. Grade 5 TRAEs occurred in 7 patients (1.5%) receiving Durvalumab 3 patients (1.3%) receiving placebo. Four deaths in the Durvalumab group (0.8%) and 2 in the placebo group (0.9%) were attributed to pneumonitis [96]. Median age was 64 years in both groups. Survival benefit with Durvalumab was observed regardless of age. OS was improved in patient aged <65 years (HR 0.66; 95% CI, 0.50–0.87) with a less significant benefit for those aged ≥65 years (HR 0.66; 95% CI, 0.60–1.05). In the univariate analyses younger age (<65 years) was identified as a favorable prognostic factor for OS and it was confirmed in the multivariable analyses [100]. Further, PFS was improved in both age groups with less significant benefit for patients aged ≥65 years. PFS ≥ 65 years: HR, 0.74 (95% CI, 0.54–1.01); <65 years: HR, 0.43 (95% CI, 0.32–0.57 [96].

An exploratory analysis of clinical outcomes from PACIFIC according to a post hoc age threshold of 70 years has been published [101]. Of the 713 randomized patients, 158 (22.2%) were aged ≥70 and 555 (77.8%) were aged <70 years. One hundred and one of 158 patients aged ≥70 (63.9%) and 375 of 555 patients aged <70 (67.6%) were randomized to Durvalumab. Among 158 patients aged ≥70 years, 56 (35.4%) were aged ≥75 years, accounting for 36.6% and 33.3% of patients in the Durvalumab and placebo arms of this group respectively. Among 555 patients aged <70 years, 164 (29.5%) were aged ≥65–70 years, accounting for 30.4% and 27.8% of patients in the Durvalumab and placebo arms of this group respectively. More patients in the over 70 age group had more comorbidities than the younger group. Pre-existing cardiovascular disorders among patients aged ≥70 years were more frequent in the Durvalumab group (Durvalumab, 75.2%; placebo, 63.2%); whereas a lower proportion of pre-existing respiratory, thoracic and mediastinal disorders was found in the Durvalumab arm (Durvalumab 58.4%; placebo, 68.4%). No clinically meaningful differences in baseline comorbidities were observed among the younger group between the two arms. Interestingly most patients had been randomized ≥14 days following completion of radiotherapy (RT), regardless of age (≥70 years, 77.2%; <70 years, 73.7%). PFS; OS and TTDM favored Durvalumab with increased clinical benefit for patients under the age of 70. At primary analysis for PFS, it was improved with Durvalumab regardless of age (median follow-up 14.5 months). In the elder group (≥70 years) median PFS was 12.3 months (95% CI, 9.2 months-NE) with Durvalumab vs. 6.1 months (95% CI, 3.6–10.9 months) with placebo (HR, 0.62; 95% CI, 0.41–0.95). Among patients aged <70 years, median PFS was 16.9 months (95% CI, 13.7 months-NE) with Durvalumab vs. 5.6 months (95% CI, 4.2–8.0 months) with placebo (HR, 0.53; 95% CI, 0.42–0.67). Further, the OS benefit favored Durvalumab regardless of age (median follow-up 25.2 months). Among patients aged ≥70 years, mOS was 29.0 months (95% CI, 21.0 months NE) with Durvalumab vs. 26.9 months (95% CI, 14.9–29.3 months) with placebo (HR, 0.78; 95% CI, 0.50–1.22). Among youngers (<70 years), median OS was not reached (NR) (95% CI, 34.7 months-NE) with Durvalumab vs. 31.0 months (95% CI, 22.9 months-NE) with placebo (HR, 0.66; 95% CI, 0.51–0.87). Moreover, TTDM favored Durvalumab in both age groups. A lower incidence of new brain lesions was found in the Durvalumab arm among patients aged ≥70 years (Durvalumab, 1%; placebo, 7%). Additionally, ORR was higher with Durvalumab: 31.9% (95% CI, 22.5–42.5) versus 17.6% (95% CI, 8.4–30.9) with placebo in the elder group; in the younger group ORR was 27.6% (95% CI, 23.0–32.5) vs. 15.4% (95% CI, 10.2–21.9). The incidence of any-grade pneumonitis/radiation pneumonitis among patients aged ≥70 years during the study was the same with Durvalumab and placebo (32.7%), unlike patients aged <70 years in which it was higher with Durvalumab (34.2%) compared with placebo (22.3%). However, G3 pneumonitis/radiation pneumonitis were more frequent among patients ≥70 years irrespective of the study treatment (Durvalumab, 7.9%; placebo, 5.5%) compared with patients <70 years (Durvalumab, 2.4%; placebo, 1.7%). No G4 pneumonitis/radiation pneumonitis events were reported. Incidence of any-grade irAEs was similar in both treatment arms in the elder group (≥70 years): 19.8% with Durvalumab and 14.5% with placebo; among patients aged <70 years irAEs were more frequent with Durvalumab (25.7%) compared with placebo (6.1%). Pneumonitis, hypothyroidism, hyperthyroidism and dermatitis/rash were the most frequent irAEs in both age groups. Among patients ≥70 years G3 immune-related pneumonitis was reported in 5.0% and 3.6% of patients with Durvalumab and placebo, respectively; among patients aged <70 years it was 1.1% and 2.2%. No G4 pneumonitis events were reported. Permanent discontinuation of Durvalumab due to AEs was more frequent in the elder group: 21.8% among patients ≥70 years vs. 13.6% among patients <70 years. The analysis of patient reported outcomes (PROs) did not indicate any detrimental effect of up to 12 months of Durvalumab versus placebo in patients aged ≥70 years, giving further evidence that it can be administered safely following CRT irrespective of age, without compromising patient QoL [101].

The effectiveness and safety of Durvalumab consolidation after CRT in a RW population of 147 patients was retrospectively evaluated and compared with 121 historical controls treated with CRT alone [102]. Median age was over 65 years in both groups; patients with age ≥ 65 years were 61.9% and 52.1% respectively [102]. With a median follow-up of 15.8 months, mOS was not reached in the Durvalumab group and 26.9 months in the historical cohort with a median follow-up of 51.5 months [102]. The 12-month OS rate was 92% versus 78.5% (HR 0.56, 95% CI: 0.37–0.85, *p* = 0.001) [102]. In the Durvalumab group most patients started consolidation after 14 days from CRT (58.7%) and 27.2% started consolidation after 42 days [102]. No OS differences were noted between patients who started immunotherapy less than 42 days and those who delayed treatment (NR in both groups, HR: 0.90, 95% CI: 0.38–2.1, *p* = 0.81) [102]. Pneumonitis of any grade occurred in 29.9% of patients and G3–5 were 6.1%, with 3 deaths attributed to pneumonitis [102]. Grade ≥3 pneumonitis were slightly more frequent in the historical control group (9.9%) [102]. Taken together these results confirm the efficacy and safety of Durvalumab consolidation after CRT as seen in PACIFIC trial also in a RW population with most patient aged over 65 years [102].

The observational study PACIFIC-R enrolled patients who received Durvalumab through EAP in order to provide the first RW data on the use and effectiveness of the PACIFIC regimen. Treatment characteristics and a preplanned analysis of real-world PFS (rwPFS), as well preliminary OS analysis (with approximately 2 y of follow-up) have been recently published [103]. PACIFIC-R included patients who received sCRT and cCRT, did not exclude patients with poor PS, included also patients with PD-L1 expression less than 1% and permitted treatment until disease progression [103]. The median age was 66.0 years at EAP entry; 21.2% and 10.4% were aged 70–75 years and above 75 years, respectively. Median OS was not reached in the full analysis set; 71.2% of the patients (95% CI, 68.8–73.6) were estimated to be alive at 24 months. Median rwPFS was 21.7 months (95% CI, 19.1–24.5), with 62.2% (95% CI, 59.6–64.6) and 48.2% (95% CI, 45.4–50.9) of the patients estimated to be alive and free of progression at 12 and 24 months, respectively. Median rwPFS was numerically similar among patients aged < 70 years and 70–75 years (22.8 versus 22.4 months respectively) and was slightly shorter among patients aged >75 years (19.2 months); it was however greater than reported by PAFICIC (16.8 months) [96]. Interestingly, only the 30.1% of the patients started Durvalumab within 42 days (and 1.2% within 14 days) of finishing RT; 14.4% and 1.0% started more than 3 months and more than 6 months after finishing RT, respectively. Durvalumab has been well tolerated; AEs of special interest (AESIs) occurred in 47.6% of patients reported, 16.5% of patients reported AESIs leading to permanent discontinuation. Pneumonitis or ILD occurred in the 17.9% of the total population; these were mainly moderate (8.4% of the total population) and only two patients (0.1%) had fatal events; the median time of onset of the first event was 2.3 months from the start of Durvalumab and led to permanent discontinuation in 9.5% of patients [103].

A meta-analysis on real-world data (RWD) investigated the efficacy and tolerability of the PACIFIC regimen [104]. Thirteen studies, including PACIFIC-R, for a total of 1.885 patients, were included. In 9 studies (69%) the median age was over 65 years, indicating that more elderly patients are treated in real life, alongside a greater proportion of PS ECOG ≥ 2 patients. The pooled 12-month OS was 90% and 12-month PFS 62%, slightly higher than those reported in the PACIFIC trial (83.1% and 55.7%, respectively) [100]. The pooled incidence of all-grade pneumonitis was 35% and 6% for ≥G3 pneumonitis; studies with older patients (median age >65 years) reported significantly higher all-grade pneumonitis rates than those with a median age ≤65 years (44%; 95% CI, 24–64% versus 15%; 95% CI, 10–21%; *p* = 0.008). As seen in PACIFIC-R [103], median time from CRT completion to Durvalumab often exceeded 42 days, seemingly without affect survival [104]. These findings make reason of a full recovery from any pulmonary toxicity after CRT before Durvalumab, especially in a frail population as elders.

Novel approaches of IT combinations with radiotherapy in LA unresectable NSCLC are under study [105].

Induction IT, with or without CT, followed by concomitant radiation represent an attractive strategy for the treatment of unresectable LA-NSCLC. IT alone appears to be intriguing for patients who cannot tolerate concomitant or sequential CT, as those with poor PS or elderly. In a recent case series, two patients over the age of 80 with stage IIIA squamous cell carcinoma and with PD-L1 TPS of 80% were treated with 7 cycles of Pembrolizumab induction followed by chest irradiation and subsequent Pembrolizumab consolidation, achieving partial response in one case and complete response in the other; in the first case Pembrolizumab was prematurely withdrawn due to pneumonitis after 4 cycles of consolidation [106]. This is approach needs to be validated in clinical trials and could allow to recover patients excluded from standard concomitant CRT, as elders. In this perspective, DUART is a phase 2, single arm, open label study (NCT04249362) designed to assess the safety and tolerability of Durvalumab following radiotherapy in patients with unresectable LA-NSCLC ineligible for chemotherapy; patients are stratified according to the dose of radiotherapy received before durvalumab: standard radiotherapy 60 Gy ± 10% or hypofractionated bioequivalent dose (BED) (cohort A); palliative radiotherapy 40 to <54 Gy or hypofractionated BED (cohort B). PS ECOG ≤ 2 is a key eligibility criterion [107].

## 10. Perioperative Immunotherapy

The use of ICIs in the perioperative setting is under investigation in several trials and is undoubtedly a promising approach [108].

The results of two phase 3 trials with adjuvant atezolizumab [109] and pembrolizumab [110] have been recently published.

Atezolizumab was tested versus placebo in the randomized phase 3 trial IMpower010 [109]. Patients with completely resected stage IB (tumor size ≥ 4 cm) to IIIA NSCLC (AJCC 7th edition) were randomly assigned (1:1) to adjuvant Atezolizumab or best supportive care after adjuvant platinum-based chemotherapy (at least one cycle). The primary endpoint DFS was tested hierarchically first in the stage II–IIIA population subgroup with PD-L1 TPS on 1% or more, then all patients in the stage II-IIIA population, and finally the ITT population (stage IB–IIIA). After a median follow-up of 32.2 months Atezolizumab demonstrated to improve DFS in the stage II–IIIA population with PD-L1 TPS ≥ 1% (median DFS NR vs. 35.3 months; HR 0.66; 95% CI, 0.50–0.88; *p* = 0.0039) and in all patients in the stage II–IIIA population (median DFS 45.3 vs. 32.3 months; HR (0.79; 0.64–0.96; *p* = 0.020). In the ITT population there was not statistically significant difference with median DFS NR versus 37.2 months, HR 0.81 (0.67–099; *p* = 0.040). In the overall population median age was 62 years; patients < 65 years were the 64% in the Atezolizumab arm and 60% in the placebo arm; patients ≥ 65 years were the 36% in the Atezolizumab arm and 40% in the placebo arm. Among patients with stage II-IIIA PD-L1 positive NSCLC, DFS favored Atezolizumab in both age groups: HR 0.67 (0.46–0.96) in those aged < 65 years; HR 0.64 (0.41–1.01) in those aged ≥65 years. DFS was better with Atezolizumab also in the overall stage II-IIIA population: HR 0.79 (0.61–1.03) in those aged <65 years; HR 0.76 (0.54–1.05) in those aged ≥65 years. Toxicity profile was consistent with that previously reported with atezolizumab monotherapy [38,56,60,111].

In the phase 3 trial PEARLS/KEYNOTE-091, 1177 patients with pathologically confirmed stage IB (≥ 4 cm in diameter), II or IIIA (AJCC 7th edition) NSCLCs with any PD-L1 expression level were randomly assigned (1:1) to Pembrolizumab 200 mg or placebo for up to 18 cycles [110]. Adjuvant chemotherapy was not mandatory. Pembrolizumab improved DFS in the general population with a median DFS of 53.6 months (95% CI 39–2 to NR) in the Pembrolizumab group versus 42.0 months (31.3 to NR) in the placebo group (HR 0.76 [95% CI 0.63–0.91], *p* = 0·0014) after a median follow-up of 35.6 months (IQR 27.1–45.5). However, Pembrolizumab failed to demonstrate a DFS advantage in the PD-L1 TPS ≥ 50% population: median DFS survival was NR in both arms (HR 0.82 [95% CI 0.57–1.18]; *p* = 0.14). Median age was 65 years (IQR 59–70) in the overall ITT population. Patients aged ≥65 years were the 52% in the Pembrolizumab arm ad 53% in the placebo arm respectively. In the ITT population, independently by PD-L1, DFS HR was 0.73 (95% CI, 0.56–0.96) for patients <65 years of age and 0.84 (CI 95%, 0.66–1.07) for those ≥65 years of age. Safety profile was similar consistent with previous studies of Pembrolizumab monotherapy in NSCLC [37,40,41,55,58,59].

No data on efficacy by age subgroups nor age-related toxicity has been presented so far for these studies.

In the neoadjuvant setting, several phase 1b and phase 2 trials tested the efficacy and safety of immunotherapy alone (PD-1/PD-L1 inhibition) demonstrating intriguing results in terms of major pathological response (MPR) rates achieving also 45% and pathological complete response (pCR) rates (7–15%) with manageable safety profile [112,113,114,115,116,117,118]. The addition of chemotherapy to immunotherapy resulted in better results in terms of MPR and cPR [119,120].

The phase 3 trial Check-Mate 816 evaluated the efficacy and safety of neoadjuvant Nivolumab plus platinum doublet chemotherapy (three cycles) as compared with platinum doublet chemotherapy alone (three cycles), before definitive surgery, in patients with resectable NSCLC, stage IB (≥4 cm) to IIIA (AJCC 7th edition), without known ALK translocations or EGFR mutations [121]. Nivolumab plus chemotherapy favored the two co-primary end points of event free survival (EFS) and pCR. After a minimum follow-up of 21 months, the median EFS was 31.6 months (95% CI, 30.2 to NR) with Nivolumab plus chemotherapy and 20.8 months (95% CI, 14.0 to 26.7) with chemotherapy alone (HR 0.63; 97.38% CI, 0.43 to 0.91; *p* = 0.005). The percentage of pCR was 24.0% (95% CI, 18.0 to 31.0) with Nivolumab plus chemotherapy and 2.2% (95% CI, 0.6 to 5.6) with chemotherapy alone (odds ratio, 13.94; 99% CI, 3.49 to 55.75; *p* < 0.001). Combination treatment was well tolerated without relevant safety signals compared to chemotherapy alone. Incidence of irAEs was low, mainly G1 or G2 events; The most common irAE of any grade with nivolumab plus chemotherapy was rash (8.5%); only 2 patients (1.1%) experienced G1 or G2 pneumonitis. Three treatment-related deaths occurred, all in the chemotherapy-alone group [121].

AEGEAN is another phase III, double-Blind, placebo-controlled trial testing Durvalumab plus chemotherapy in neoadjuvant setting, followed by adjuvant Durvalumab in patients with resectable stages II-III NSCLC (NCT03800134); elderly patients with PS ECOG 0–1 were allowed [122]. Enrollment has been concluded and the results are still attended to the moment of the writing of this paper.

## 11. Case Reports

The efficacy of ICIs monotherapy was highlighted also in several case reports in patients aged over 70 years.

Remarkable radiological responses have been described even after a few cycles of single agent immunotherapy, including a complete response after 5 courses of Pembrolizumab in an 84-year-old man with SCC and PD-L1 TPS ≥ 80% [123]. Another complete and durable response (over two years) has been described with Durvalumab in a 77-year-old man with stage IV SCC (PD-L1 TPS 60%); interstitial lung disease was not considered as a limiting factor [124]. Preexisting interstitial pneumonia was not considered as an exclusion criterion even for a 72-year-old patient affected by pleomorphic carcinoma (PD-L1 TPS 65%) treated with Pembroluzumab as second-line treatment obtaining a remarkable tumor shrinkage and a prolonged response even after two years after treatment discontinuation; Pembrolizumab was permanently stopped due to hands arthralgia after ten months and the patients did not experienced any exacerbation of interstitial pneumonia [125]. Permanent discontinuation of ICIs due to AEs is frequently associated with durable response even after several months from the interruption [125,126,127].

Most relevant responses to ICIs have been often reported in elderly patients with high levels of PD-L1 TPS [123,124,125,126,128,129,130].

Interestingly, ICIs rechallenge has been associated to response in elderly patients. This is the case of a 76-year-old man with PD-L1 negative adenocarcinoma who received Nivolumab as second-line treatment after Carboplatin-Pemetrexed, obtaining PR; at relapse he was treated with Atezolizumab achieving complete response at the 6-months scan; no irAEs were reported [131]. Similarly, a 79-year-old woman with TP53-mutated SCC and PD-L1 TPS of 30% relapsed after sCRT and was initially treated with Nivolumab; at disease progression carboplatin-paclitaxel was resumed, obtaining progressive disease and Atezolizumab was therefore initiated as fourth line with an objective PR documented at first evaluation; no irAEs were reported [132]. In another report a 72-year-old Japanese male with advanced adenocarcinoma relapsed after first-line PD-1 inhibitor (within clinical trial); he was subsequently treated with 4 more lines of chemotherapy after which ICI was reintroduced (Nivolumab) obtaining tumor shrinkage [133]. A 75-year-old man with adenocarcinoma and PD-L1 TPS 25–49% was initially treated with Pembrolizumab obtaining PR but it was prematurely discontinued due to tubulo-interstitial nephritis; at disease progression he was rechallenged with Atezolizumab obtaining a response which has been sustained for more than 15 months without recurrence of nephritis [134].

Several irAEs of special interest have been described in elderly patients treated with PD1/PD-L1 axis inhibitors and are summarized in Table 2. They included severe cutaneous adverse reactions [135,136,137,138,139,140], secondary sclerosing cholangitis [141], colangiopathy [142,143], immune-related hepatitis [144,145], type 1 diabetes [146,147,148], hypophysitis [149], primary adrenal insufficiency [150], myasthenia gravis and myopathy [151,152,153,154], myocarditis [154,155,156,157], severe heart failure [158], eosinophilic vasculitis and arteritic anterior ischemic optic neuropathy [159], blue toe syndrome due to peripheral vasculitis [160], Vogt–Koyanagi–Harada Disease-like Uveitis [161], acute interstitial nephritis [134,162], fulminant Goodpasture’s disease [163], neuromyelitis optica [164], isolated optic neuritis [165], encephalitis [166], hypereosinophilia [167], severe neutropenia [144,168,169,170], immune thrombocytopenia [171], autoimmune hemolytic anemia and hemophagocytic lymphohistiocytosis [172].

## 12. Discussion

Lung cancer is the third most frequent cancer and the first cause of cancer death worldwide [1]; most patients are diagnosed in advanced age, with median age at diagnosis of 71 years and a peak incidence in the age group between 65 and 74 years; 36.2% of cases are diagnosed in patients aged 75 or older [4]. The treatment of elderly patients is challenging due to the higher risk of adverse events determined by comorbidities; deteriorations in physical, organ, and cognitive functions; and reduced social support. Moreover, elderly patients are underrepresented in clinical trials. All these aspects give a reason for the under-treatment of geriatric patients in clinical practice, mainly with palliative rather than curative intent.

Immunotherapy represents a great advance in the treatment of NSCLC, given its efficacy over standard chemotherapy and an improved quality of life (QoL). This latter aspect makes ICIs an intriguing choice also for the treatment of elderly patients.

However, the effectiveness of ICIs has been questioned in older adults, given the so called immunosenescence, a phenomenon that refers to the aging process of the immune system involving both adaptive and innate immunity and which accounts for a reduced response to cancer cells or other stimuli, as described by Ferrara et al. [14].

In this review, we analyzed the most important clinical trials testing ICIs, most of which have allowed their use in clinical practice, focusing on differences between age groups. We also considered the RW setting studies published so far.

In the second-line setting, Nivolumab was demonstrated to improve survival compared to Docetaxel in patients with squamous NSCLC aged 65 to 74 years old in the CheckMate trial 017 (HR 0.56; 95% CI, 0.34–0.91) [34] and in those with nonsquamous histology aged ≥65 years (HR 0.63, 95% CI, 0.45–0.89) in the CheckMate 057 trial [35]. The same advantage was not noted in patients aged ≥75 years with squamous histology [34] and was less evident in the same age group with nonsquamous NSCLC [35], even if they represented only the minority of enrolled patients, respectively the 11% and 7% of the overall population in both studies. Further, in the pooled analysis of CheckMate 017 and CheckMate 057, Nivolumab showed an advantage in survival after 5 years in patients over 65 years (HR 0.71), but this benefit was lost when considering people aged 75 years or more (HR 1.19) [36]. These results were independent from PD-L1 TPS. The phase 2 trial KEYNOTE-010 demonstrated the superiority in OS of Pembrolizumab compared to Docetaxel in NSCLC with PD-L1 TPS ≥ 1%, also in patients aged ≥65 years (HR 0.80, 95% CI 0.60–1.01). Atezolizumab was associated with better survival when compared to Docetaxel independently of PD-L1 in the ITT population and in patients aged ≥65 years, both in the phase 2 trial POPLAR [56] and in the phase 3 trial OAK [38]. However, considering the long-term survivors (≥4 years) aged 65 years old or more, their proportion was similar across the two treatment arms in both studies: 7% of the ITT in the Atezolizumab arm and 4% in the Docetaxel arm in POPLAR; 6% in the Atezolizumab arm and 4% in the Docetaxel arm in OAK [57].

No safety signals emerged in these clinical trials considering the elderly population. These results taken together confirm the efficacy of the PD1/PD-L1 inhibition in previously treated advanced NSCLC, independently from histology and PD-L1 expression also in the elderly population, with 65 years old as cut-off.

Moving to the first-line setting, Pembrolizumab, Atezolizumab, and Cemiplimab were demonstrated to improve survival in patients aged over 65 years compared to standard platinum-based chemotherapy. Pembrolizumab showed better PFS and OS in patients with PD-L1 TPS ≥ 50% and wild type for EGFR and ALK, in the overall population, in patients < 65 years (HR 0.60; 95% CI, 0.38–0.96) and in those ≥65 years (HR 0.64; 95% CI, 0.42–0.98) [39]. A pooled analysis using data from KEYNOTE-010, KEYNOTE-024, and KEYNOTE-042 explored the efficacy and safety of Pembrolizumab compared to standard chemotherapy in the elderly population (aged ≥75 years) [41]. All patients were PS ECOG of 0–1, without clinically relevant conditions. Among patients aged ≥75 years, the median age was 77 years (range, 75–90). Pembrolizumab improved OS, regardless of age and PD-L1 TPS. This advantage was maintained among the elderly population with PD-L1 TPS ≥ 50% in the first-line setting. No new safety signals were reported. Pembrolizumab determined a lower rate of TRAEs, G3 TRAEs, and SAEs compared with chemotherapy among the elderly patients. Grade 3–4 irAEs occurred in the 9.4% of elderly patients, compared to the 6.8% in younger patients [41]. Cemiplimab demonstrated a better OS ad safety profile vs. standard platinum-doublet chemotherapy in both age groups; no safety evaluation was reported by age [43]. Further, Atezolizumab demonstrated to be better than standard platinum-based chemotherapy (PD-L1 ≥ 1% on TC or IC), in the overall population and in patients aged 65–74 years (HR 0.63; (95% CI 0.34–1.19), with a reduction of the risk of death in patients older than 74 of 21%, even if they accounted only for 11.2% of the global population (HR 0.79; 95% CI 0.18–3.56) [60]. However, at the updated OS analysis, this advantage was confirmed among patients aged 65–74 years (HR 0.78; 95% CI 0.45–1.36), but not in those aged over 74 (HR 1.03, 95% CI 0.31–3.48) [42].

A meta-analysis of randomized trials with ICIs evaluated the efficacy of ICIs between younger and older patients [173]. A total of 5265 patients from 8 phase 3 and one phase 2 randomized trials in different malignancies were included: melanoma (5 trials), NSCLC (2 trials), prostate cancer (1 trial), and renal cell carcinoma (1 trial). Three trials tested Ipilimumab, 4 trials Nivolumab, 1 trail Pembrolizumab, and 1 trial Tremelimumab. With an age cut-off of 65–70 years, the pooled HR for OS between younger patients showed significant differences between ICIs and controls (HR, 0.75; 95% CI, 0.68–0.82; *p* < 0.001). Moreover, between older patients, ICIs significantly improved OS in comparison with controls (HR, 0.73; 95% CI, 0.62–0.87; *p* < 0.001). There was no statistically significant difference between younger and older patients concerning the pooled HRs for OS (*p* = 0.96). A total of three anti-PD-1 trials comprising 1394 patients were available for the analysis of HRs for PFS. With a cut-off age of 65 years, PFS in the younger group favored ICIs therapy (HR 0.58; 95% CI, 0.40–0.84; *p* = 0.004); PFS was not significantly different in the older group, but there was a trend toward improvement with ICIs (HR, 0.77; 95% CI, 0.58–1.01; *p* = 0.06) [173].

The IPSOS study is the first phase III trial specifically designed to compare first-line immunotherapy (Atezolizumab) versus single-agent chemotherapy in a frail population: patients not eligible for a platinum-containing regimen due to poor PS (ECOG ≥2) or to age (≥70 years) [44]. The survival benefit for Atezolizumab was observed in the group of patients under the age of 70 years (HR 0.75, 95% CI 0.49–1.14) and in the group of patients aged 71 to 79 years (HR 0.68, 95% CI 0.49–0.94), but not in patients aged 80 or over (HR 0.97, 95% CI 0.66–1.44). IPSOS is the first randomized study to show that first-line Atezolizumab improves OS in a poor-prognosis population with no EGFR and ALK alterations, regardless of histology, PD-L1 status, and ECOG PS with no new safety signals identified, while maintaining QoL.

From the results listed above, considering both the first-line and pretreated patients, single-agent ICIs are effective also in patients aged 65 years old or more, but a lower benefit could be assumed for very elderly patients, although this population was not adequately represented in clinical trials.

Moving to the ICIs plus chemotherapy combination, the addition of Pembrolizumab to platinum-based chemotherapy was demonstrated to improve survival in both age groups, with a cut-off of 65 years, in the KEYNOTE-189 [62] and in the KEYNOTE-407 [46] trials. Cemiplimab combined with chemotherapy showed a less meaningful survival advantage in older adults than youngers, even if it was numerically better than with chemotherapy alone [47]. Similarly, a less depth of benefit was noted across age subgroups in the phase 3 trial IMpower131 with Atezoluzumab in squamous NSCLC, although elderly patients (aged 74–85 years) were only the 11.4% [48,63]. Further, in the IMpower 150 phase 3 trial, which tested the combination of ABCP versus BCP in nonsquamous NSCLCs, elders were underrepresented: patients aged 65–74 years were 37.2% and those aged 75–84 years were 8.2%; patients older than 85 years were only 0.8%. Moreover, in this trial, the OS benefit was less evident in advanced age: HR 0.72 (95% CI, 0.54–0.97) among patients aged 65–74 years and HR 0.97 (95% CI, 0.58–1.62) among patients aged 75–84 years [49]. The safety profile was consistent with the ones of each drug in these combinations, and the rate of irAEs was as expected.

Another first-line strategy is the dual immune checkpoint inhibition with anti-CTLA4 combined with anti-PD1/PDL1 antibodies. In the open-label phase 3 trial CheckMate 227, the combination of Nivolumab plus Ipilimumab resulted in a longer OS than did chemotherapy, independently of the PD-L1 TPS [68]. However, patients aged ≥75 years accounted only for 9.9% of the total population in the experimental arm, and those aged 65–75 years 37.6%. The OS benefit seems to get lost, advancing with age in the PD-L1 positive group: HRs were 0.91 among patients aged 65–74 years and 0.92 among patients aged 75 years or more. However, in the PD-L1 negative group, HRs were 0.49 among patients aged 65–74 years and 0.75 among patients aged 75 years or more. More patients aged 65 and over were enrolled in the PD-L1 positive group (387 vs. 168) [68]. Treatment-related SAEs of any grade were more common with ICIs combination (24.5% vs. 13.9%), as well as TRAEs leading to discontinuation (18.1% vs. 9.1%). Skin reactions and endocrine events were the most common immune-related TRAEs.

A pooled analysis evaluated the safety of first-line Nivolumab plus Ipilimumab in a large population from three first-line studies [69] CheckMate 227 part 1 [68], CheckMate 817 cohort A [70,71,72], and CheckMate 568 part 1 [73]. In the pooled population of 1255 patients, the median age was 65 years and patients aged 75 years or older were only 14%. Grade 3–4 TRAEs occurred in 34% of patients and discontinuation of any regimen component owing to TRAEs in 21%. More patients aged ≥75 years had an ECOG PS of 1, compared to the general population. TRAEs of grade 3 and 4 were numerically more frequent in the elder group (44% vs. 34%), as well as TRAEs leading to discontinuation (29% vs. 21%). Serious TRAEs were also numerically more frequent among elders (29% vs. 23%). The incidence of any grade irAEs was comparable with the overall population. This pooled data provided the evidence of a generally consistent and manageable safety profile across age groups. As with the overall population, most non-endocrine irAEs resolved, suggesting these events are manageable in the older age group. Patients aged 75 years or older who discontinued Nivolumab plus Ipilimumab treatment owing to TRAEs also had OS bene fits similar to the overall pooled population.

The CheckMate 9LA trial evaluated Nivolumab plus Ipilimumab combined with histology-based, platinum doublet chemotherapy for two cycles followed by ICIs combination up to 2 years, versus chemotherapy alone (up to four cycles or Pemetrexed maintenance in nonsquamous NSCLC), demonstrating the superiority of the experimental arm in survival. The treatment was overall well tolerated and the majority of grade 3/4 TRAEs in the combination arm occurred during the first two cycles, corresponding to the duration of the limited course of platinum-doublet chemotherapy, thus suggesting that they were mostly related to the association of chemotherapy. Most enrolled patients were under the age of 65: 354 compared to 295 of those aged 65–75, and only 70 patients were older than 75 years. The survival advantage is lost for patients older than 75 years: unstratified HR 1.04 1.04 (95% CI, 0.63–1.72) [51]. No conclusions can be drawn about the group of elderly patients, given the small sample size. The only two rounds of chemotherapy could represent an advantage in subjects with reduced organ reserve, especially bone marrow and kidney, such as the elderly patients.

The lack of representativeness of elderly patients in clinical trials is certainly a limit when considering these data and trying to evaluate them with a clinical practice perspective. Several real-world (RW) retrospective and observational studies evaluated the activity of ICIs also in the elderly population.

The large retrospective study by Takamori S and coll. showed that chronological age does not appear to impact the survival benefit of ICIs in patients with stage IV NSCLC [87].

The efficacy of Nivolumab monotherapy in the second line was confirmed in the Italian EAP studies in both squamous [76] and nonsquamous NSCLC [78], with a less depth of benefit in survival within the elderly population in squamous NSCLC over the age of 75 years [76]. Further, in the French EAP, age was not a predictor of survival [79].

Other retrospective studies confirmed the feasibility of ICIs monotherapy in the elderly population, without affecting the tolerability [80,81]. Pembrolizumab monotherapy in the first-line setting in patients with PD-L1 TPS ≥ 50% resulted in a similar OS benefit between elderly and younger patients, with a similar toxicity profile [83,84]. Moreover, in a large retrospective study on 2302 patients with advanced NSCLC who received ICIs, survival was not significantly different for patients aged over 75 years even if they were only 9% [86]. Interestingly, data from the REMSAIC registry demonstrated a higher incidence of irAEs grade ≥ 2 between the elders (33% versus 25%, *p* = 0.035) with a difference in organs affected: the incidence of skin toxicities was much higher in the old group (*p* 0.007), while the endocrine toxicities were more frequent in the young group (*p* = 0.044). Even if the difference was not significant, elders experienced a higher incidence of multiple irAEs of grade ≥ 2 per individual and a slightly higher discontinuation rate due to irAEs [91].

In the large analysis from the SEER database on 1256 patients aged 65 years old or more, multiple comorbid conditions, squamous histology, a history of non-platinum doublet therapy, recent radiotherapy, and a shorter time from the initial diagnosis to treatment initiation were factors associated with increased risk of death in multivariable survival analyses [85].

Results of the ELDER study (a prospective observational study) are of considerable interest and highlight the utility of a geriatric assessment before starting the treatment of elderly patients [92]. The geriatric assessment identified the 95% of patients with positive G8 screening (the frail subgroup) at the first study visit, also including those who could have been considered fit on the basis of performance status evaluation, confirming that PS alone is not reliable to assess the functional level of older patients, as previously described [174]. The incidence of any grade irAEs and G3–5 irAEs were slightly higher in the older cohort, but not significantly different. However, the management of irAEs may be more challenging in elders, due to the prolonged use of systemic steroids, which may lead to other effects, such as decompensation of pre-existing diseases. A positive G8 screening was a predictor of hospital admission, but only 32% of the admission of frail patients were treatment-related. Moreover, a positive G8 screening was also a predictor of death, together with polypharmacy and a higher comorbidity score [92]. It can be inferred that geriatric assessment is more efficient than PS in identifying frail patients at higher risk of death and hospital admission. Frail patients should be carefully followed by a geriatrician in order to take corrective interventions, and this specialist should be part of the multidisciplinary team.

Retrospective RW studies have been reported also for combination regimens. Globally, they confirmed the efficacy of chemotherapy plus ICIs and at the same time highlighted a slight increased risk of toxicity in the elderly population [93,94], with a higher rate of discontinuation of all treatment components due to AEs [93].

No real-world data have been published so far on the double ICIs combination alone or with chemotherapy.

The only two rounds of chemotherapy in the CheckMate 9LA schedule could represent an advantage in subjects with reduced organ reserve, especially bone marrow and kidney, such as the elderly. However, this perspective should be considered carefully, given the lower efficacy of this combination among elderly patients, even if the sample size was very small.

Considering the locally advanced disease, the survival benefit with Durvalumab in the PACIFIC trial was observed regardless of age [96]. Further, the exploratory analysis according to a post hoc age threshold of 70 years demonstrated an advantage for Durvalumab versus Placebo irrespective of age, even if survival was higher in those under the age of 70 years. Grade 3 pneumonitis/radiation pneumonitis was more frequent among patients ≥70 years, irrespective of the study treatment, and the incidence of any-grade irAEs was similar in both treatment arms in the elder group. Moreover, the analysis of PROs did not indicate any detrimental effect of up to 12 months of Durvalumab. This data indicate that Durvalumab is associated with treatment benefit and a manageable safety profile, also in patients aged 70 years and over without affecting PROs, suggesting that eligible patients should not be excluded from the PACIFIC regimen [101]. In the observational study PACIFIC-R, the median age was 66.0 years at EAP entry, with 21.2% patients aged 70–75 years and 10.4% above 75 years. Median rwPFS was numerically similar among patients aged <70 years and 70–75 years (22.8 versus 22.4 months respectively) and was slightly shorter among patients aged >75 years (19.2 months) [103]. A meta-analysis on real-world data including 1885 patients showed that more elderly patients are treated in real life, alongside a greater proportion of PS ECOG ≥ 2 patients. Studies with older patients (median age >65 years) reported significantly higher all-grade pneumonitis rates than those with a median age ≤65 years. As seen in PACIFIC-R, the median time from CRT completion to Durvalumab often exceeded 42 days, seemingly without affecting the survival regimen [102,104]. These findings make a reason for a full recovery from any pulmonary toxicity after CRT before Durvalumab, especially in a frail population, such as elders.

Novel approaches of IT combinations with radiotherapy in unresectable LA NSCLC are desirable, and in this perspective induction IT, with or without CT, followed by concomitant radiation represent an attractive strategy also in elderly patients or those with poor PS.

In the perioperative setting, data from randomized clinical trials showed comparable results in patients under the age of 65 years and in those aged 65 years old or more, with a tolerability consistent with the well-known toxicity profile of ICIs; however, AEs were not described by age groups.

Taken together, results from randomized clinical trials and RW studies confirm the efficacy of ICIs in patients aged equal or more than 65 years. Single-agent ICI in older cancer patients was not associated with a higher incidence of high-grade immune toxicity, but the impact of irAEs may be more challenging due to the comorbidity burden and reduced organ function. However, the treatment of elder patients, aged 70 years or more, should be carefully evaluated, especially when considering those aged more than 75 years, given the potential loss of clinical benefit with advancing age, associated with an increased risk of toxicity. This is more evident when considering ICIs combinations. Patients with SCC aged 75 years old or more did not benefit from second-line Nivolumab (HR 1.85; 95% CI, 0.76–4.51) in the CheckMate 017 trial, even if the they were only 11% of total population [34]. This was not observed in those with nonsquamous histology with a 10% reduction in the risk of death noted with Nivolumab, as compared with docetaxel: HR 0.90 (95% CI, 0.43–1.87) [35]. Further, the pooled analysis of these two trials highlighted a loss of benefit in people aged 75 years or more (HR 1.19), despite the small sample size (72 patients) [36]. In the first-line setting, Atezolizumab did not show a survival benefit compared to chemotherapy in patients aged over 74 with high PD-L1 expression and WT for EGFR or ALK (25.6 months versus NE; HR 1.03, 95% CI 0.31–3.48) [42]. Considering the first-line setting, Atezolizumab added to the combination BCP resulted in a loss of benefit in terms of survival when compared to BCP among patients aged 75–84 years, even if it was not detrimental: HR; 0.97 (95% CI, 0.58–1.62), mOS 16.7–14.1 months [49]. Even with the combination Ipilimumab-Nivolumab, the survival benefit tends to get lost with age compared to standard platinum-based chemotherapy, regardless of PD-L1 TPS level (Table 1) [68]. In the CheckMate 9LA trial, most enrolled patients were under the age of 65, with those aged 65–75 representing 41% and only those older than 75 years representing only 11%. The survival benefit is maintained in the first two groups, while it is lost for patients older than 75 years with a mOS of 8.5 months compared to 11.5 months obtained with chemotherapy alone and an unstratified HR of 1.04 1.04 (95% CI 0.63–1.72) [51]. However, from this point of view, a definitive conclusion cannot be drawn, especially from randomized clinical trials, given the poor representativeness of the sample among patients over 70 or 75 years old, which often barely reach 11% of the total population. More in-depth assessments will come from clinical practice and observational studies.

Some randomized clinical trials specifically designed for elderly patients will provide more evidence for the treatment of this population. ELDERLY (NCT03977194) is a phase 3 randomized trial testing the combination of Carboplatin plus weekly paclitaxel alone or in combination with Atezolizumab in patients aged 70 to 89 years. MILSES-5 (NCT03975114) is a phase 2 randomized trial comparing standard chemotherapy followed by Durvalumab at progression versus Durvalumab, followed by chemotherapy at progression versus combination immunotherapy with Durvalumab plus Tremelimumab, followed at progression by chemotherapy. An interesting study is the phase 2 randomized DURATION trial, in which patients aged ≥70 years and/or with a Charlson-Comorbidity-Index > 1 and/or ECOG PS > 1 not eligible to platinum-doublet are divided into two groups: those with CARG-Score ≤ 3 are randomized to Carboplatin plus nab-Paclitaxel or the same schedule for two cycles, followed by Durvalumab maintenance; those with CARG-score > 3 are randomized to vinorelbine or gemcitabine for two cycles, followed by Durvalumab versus chemotherapy alone; the primary end point is the safety (incidence of grade≥ 3 TRAEs).

Several clinical factors have been explored in search of predictors of immunotherapy effectiveness. Among them, sex is emerging as an important variable to be to be taken into account when considering immunotherapy, alongside age. Female sex is associated with stronger innate and adaptive immune responses than men [175]. A stronger anti-tumor immune response in females may be responsible for the improved cancer-related outcomes. Two large retrospective studies demonstrated a better survival among females [176,177], especially when considering nonsquamous NSCLC [177]. Data from single-agent ICIs studies are disappointing, but globally it seems that ICIs may result more effectively in men than women; perhaps because tumours in men are potentially more immunogenic given a higher mutational burden [178]. Conversely, a systematic review and meta-analysis of randomized clinical trials showed that a greater improvement in OS and PFS was observed in female patients than in male patients with combination chemoimmunotherapy as a first-line therapy [179]. However, available evidence is not strong enough to influence treatment choices; an insufficient number of studies analyze data by sex in a prospective and pre-planned manner. Further prospective studies sufficiently powered are needed to characterize better the effect of sex on immunotherapy efficacy in patients with NSCLC [178]. No meaningful data on gender differences in elderly patients affected by NSCLC and treated with immunotherapy have been published so far to the best of our knowledge.

A step forward that oncologists should take in their approach to the geriatric patient with NSCLC is the proper clinical evaluation of the older patient, and this should be carried out through the execution of a comprehensive geriatric assessment (CGA) with the aim of identifying frail and vulnerable patients through the exploration of different health domains, such as psychological status, cognitive functions, functional status, nutritional status, comorbidities, polypharmacy, and social support. Treatment decisions for older patients can be difficult due to the complexity of cancer itself (e.g., difficulties in diagnosis and staging, multidisciplinary approach, and medical or surgical complications) and to the complexity of the individual patient (e.g., comorbidities and polypharmacy with the need to assess drug interactions, and nutritional deficiencies complicated by cancer). Given the multidimensional evaluation provided by a CGA, it can be time-consuming and therefore missed by oncologists at the first evaluation of the NSCLC patient. However, a correct initial classification of the patient would save time later, allowing for tailoring the therapy for the subject and reducing the impact in terms of toxicities and QoL [180]. It has been shown that CGA improves treatments adherence and tolerance [181]. Pre-treatment assessment is encouraged by the guidelines, and multiple screening tools have been tested and validated in this setting [182]. The G8 is one of the most commonly used screening tools, given its usability and ease of completion [183]. Patients who test positive with G8 should receive a CGA [182]. The G8 screening tool demonstrated its efficacy in the ELDERS study, in the context of immunotherapy in NSCLC, showing that also patients who could be considered fit on the basis of PS evaluation tested positive at G8 and were therefore considered as frail [92]. In another Japanese retrospective study on 434 advanced NSCLC patients who received ICIs, 100 patients aged ≥75 years were identified; G8 positively correlated with survival, since the median survival time was longer in the high modified G8 (≥12.0) group than in the low modified G8 (≤11.0) group (18.7 vs. 8.7 months; *p* = 0.02) [184]. These are the only two observational studies exploring the role of G8 in the context of NSCLC and immunotherapy in this disease. No prospective trial employing the G8 instrument has been presented so far to the best of our knowledge.

Elderly patients are often treated with multiple drugs due to multimorbidity. Polypharmacy, defined as five or more prescriptions [185], has been shown to be associated with frailty and with increased risk of death in patients treated with immunotherapy [92]. A Japanese retrospective study evaluated the efficacy of ICIs among 157 patients aged ≥65 years with advanced NSCLC; the prevalence of polypharmacy, defined as ≥5 medications, was 59.9%. The mOS in patients with and without polypharmacy was 9.5 and 28.1 months, respectively (*p* < 0.001). Multivariate analysis revealed a marked associations between polypharmacy and OS. Moreover, it was associated with a higher rate of unexpected hospitalizations during ICI treatment (59.6% vs. 31.7%, *p* < 0.001), but was not associated with irAEs [186]. Cortellini e coll. presented the outcomes analysis according to concomitant baseline medications (prior to ICI initiation) with putative immune-modulatory effects in a large cohort of 950 patients with metastatic NSCLC with a PD-L1 expression ≥50%, receiving first-line Pembrolizumab monotherapy; 595 patients treated with chemotherapy represented the control cohort [187]. No association with clinical outcomes was found according to baseline statin, aspirin, β-blocker, and metformin within the Pembrolizumab cohort. On the multivariable analysis, antibiotics were a strong predictor of worse OS and PFS in the Pembrolizumab, but not in the chemotherapy cohort; corticosteroids were associated with shorter PFS and OS in both cohorts; proton pump inhibitors (PPI) were associated with worse OS with Pembrolizumab and chemotherapy [187]. A drug-based prognostic score with the above-mentioned drugs was developed and validated [188] and demonstrated a good ability to stratify NSCLC patients candidate to Pembrolizumab monotherapy [189]. More recently, a review on retrospective studies exploring the role of angiotensin-converting-enzyme inhibitor (ACEi), angiotensin receptor blockers (ARBs), and aspirin did not significantly impact ICI efficacy or worsen toxicity, even if most studies are small and underpowered [190]. Larger, prospective studies are needed to address the role of concomitant medications during ICIs therapy and identify drugs that it would be better to avoid during immunotherapy, given the burden of older patients treated in real life.

**Figure 1:**
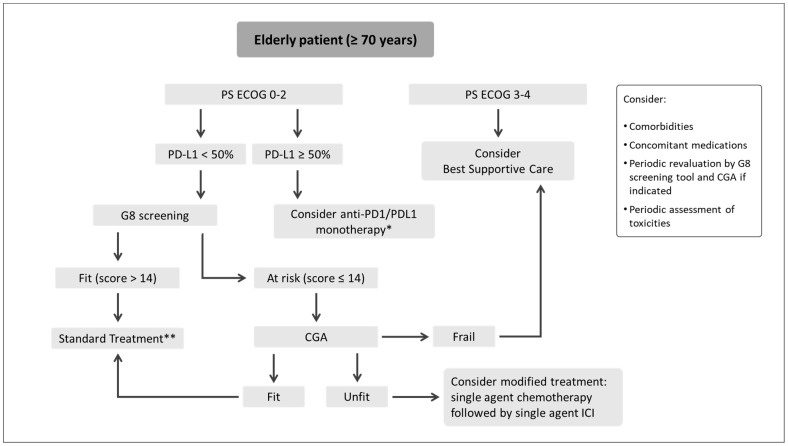
Proposal for a decision algorithm in elderly patients with NSCLC. * G8 screening should be done also in patients with PD-L1 TPS ≥ 50%. Combination therapy should be considered with due caution, given the effectiveness of single agent immunotherapy. Best supportive care alone could be an option for patients found frail at CGA. ** Chemotherapy plus immunotherapy should be considered. The association of double ICIs plus chemotherapy should be carefully evaluated, given the loss of survival benefit in patients older than 75 years. Patients with PS ECOG 2 and PD-L1 TPS < 50% should be considered for non-platinum single-agent chemotherapy followed by single-agent ICI.

The drafting of a therapeutic algorithm in the elderly patient is made particularly difficult by the many variables to be taken into account, such as geriatric assessment, comorbidities, and polypharmacy, as well as the clinical and biological variables that guide the therapeutic choice in the NSCLC. Figure 1 is a proposal for a decision algorithm for elderly patients (older than 70 years), based on the evidence available so far from randomized clinical trials and retrospective studies.

## 13. Conclusions

The use of ICIs should not be discouraged in elderly patients, given their proven effectiveness even in subjects older than 65 years. However, particular caution should be taken in very elderly patients, given the likely reduction of treatment efficiency with increasing age and a tendency to increased risk of adverse reactions, especially with combinations of anti-PD1 and anti-CTLA4. A preliminary assessment of the elderly patient with tools, such as G8, should be ensured in order to screen patients who may need a CGA, avoiding unnecessary toxicity in frail elderly patients. Polypharmacy is a prognostic factor to be considered in older patients with advanced NSCLC candidate to treatment with ICI, given the potential detrimental effect of certain drugs, such as antibiotics and corticosteroids. Due to the small number of elderly patients in registration trials, randomized clinical trials specifically designed for elderly patients are desirable.

## Figures and Tables

**Table 1:** Overall survival by age in clinical trials and pooled analyses investigating ICIs in advanced NSCLC.

Study [Ref]	Drug	Setting	Population	Age (Years)	Patients n.	OS HR (95% CI)
CM 017 [34]	Nivolumab vs. Docetaxel	2L	NSCLC squamous, PD-L1 any	<65	152	0.52 (0.35–0.75)
				≥65–75	91	0.56 (0.34–0.91)
				≥75	29	1.85 (0.76–4.51)
CM 057 [35]	Nivolumab vs. Docetaxel	2L–3L	NSCLC nonsquamous PD-L1 any	<65	333	0.81 (0.62–1.04)
				≥65–75	200	0.63 (0.45–0.89)
				≥75	43	0.90 (0.43–1.87)
Pooled CM 017—CM 057 [36]	Nivolumab vs. Docetaxel	2L	NSCLC, PD-L1 any	<65	491	0.66
				≥65–75	363	0.71
				≥75	72	1.19
KN-010 [37]	Pembrolizumab vs. Docetaxel	≥2L	NSCLC, PD-L1 ≥ 1%	<65	604	0.62 (0.52–0.65)
				≥65	429	0.80 (0.64–1.01)
OAK [38]	Atezolizumab vs. Docetaxel	2L–3L	NSCLC, PD-L1 any	<65	453	0.80 (0.64–1.00)
				≥65 yr	397	0.66 (0.52–0.83)
KN-024 [39]	Pembrolizumab vs. standard platinum doublet chemotherapy	1L	NSCLC, PD-L1 ≥ 50%	<65	141	0.60 (0.38–0.96)
				≥65	164	0.64 (0.42–0.98)
KN-042 [40]	Pembrolizumab vs. standard platinum doublet chemotherapy	1L	NSCLC, PD-L1 ≥ 1%	<65	707	0.81 (0.60–1.08)
				≥65	567	0.82 (0.66–1.01)
Pooled KN-010—KN-024—KN-042 [41]	Pembrolizumab vs. standard chemotherapy	≥1L	NSCLC, PD-L1 ≥ 1%	<75	2348	0.76 (0.69–0.84)
				≥75	264	0.76 (0.56–1.02)
Pooled KN-010—KN-024—KN-042 [41]	Pembrolizumab vs. standard chemotherapy	≥1L	NSCLC, PD-L1 ≥ 50%	<75	1214	0.67 (0.57–0.78)
				≥75	132	0.40 (0.25–0.64)
Pooled KN-010—KN-024—KN-042 [41]	Pembrolizumab vs. standard chemotherapy	1L	NSCLC, PD-L1 ≥ 50%	<75	811	0.71 (0.59–0.87)
				≥75	93	0.41 (0.23–0.73)
IMpower110 [42]	Atezolizumab vs. standard platinum doublet chemotherapy	1L	NSCLC, PD-L1 TPS ≥ 50%/TC ≥ 10	<65	102	0.72 (0.44–1.19)
				65–74	80	0.78 (0.45–1.36)
				>74	23	1.03 (0.31–3.48)
EMPOWER-Lung 1 [43]	Cemiplimab vs. standard platinum doublet chemotherapy	1L	NSCLC, PD-L1 ≥ 50%	<65	157	0.66 (0.44–1.0)
				≥65	126	0.48 (0.30–0.66)
IPSOS [44]	Atezolizumab vs. single agent Gemcitabine or Vinorelbine	1L	NSCLC, PD-L1 any	<70	123	0.75 (0.49–1.14)
				70–79	190	0.68 (0.49–0.94)
				≥80	140	0.97 (0.66–1.44)
KN-189 [45]	Pembrolizumab + Platinum-Pemetrexed vs. Platinum-Pemetrexed	1L	NSCLC, PD-L1 any	<65	312	0.49 (0.37–0.65)
				≥65	304	0.72 (0.54–0.97)
KN-407 [46]	Pembrolizumab + Carboplatin-(Nab)Paclitaxel vs. Carboplatin-(Nab)Paclitaxel	1L	NSCLC, PD-L1 any	<65	254	0.52 (0.34–0.80
				≥65	305	0.74 (0.51–1.07)
EMPOWER-Lung 3 [47]	Cemiplimab + Platinum doublet chemotherapy vs. Platinum doublet chemotherapy	1L	NSCLC, PD-L1 any	<65	278	0.57 (0.40–0.81)
				≥65	188	0.88 (0.56–1.37)
IMpower131 [48]	Atezolizumab + Carboplatin-(Nab)Paclitaxel vs. Carboplatin-(Nab)Paclitaxel	1L	NSCLC squamous, PD-L1 any	<65	326	0.89 (0.68–1.15)
				65–74	279	0.84 (0.63–1.13)
				75–84	77	0.74 (0.45–1.23)
IMpower150 [49]	Atezolizumab + Carboplatin-Paclitaxel + Bevacizumab vs. Carboplatin-Paclitaxel + Bevacizumab	1L	NSCLC nonsquamous, PD-L1 any	<65	441	0.83 (0.65–1.04)
				65–74	281	0.72 (CI, 0.54–0.97)
				75–84	72	0.97 (0.58–1.62)
CM 227 [50]	Nivolumab + Ipilimumab vs. standard platinum doublet chemotherapy	1L	NSCLC, PD-L1 ≥ 1%	<65	406	0.72 (0.55–0.93)
				65–74	306	0.85 (0.64–1.13)
				≥75	81	0.95 (0.56–1.62)
CM 227 [50]	Nivolumab + Ipilimumab vs. standard platinum doublet chemotherapy	1L	NSCLC, PD-L1 < 1%	<65	205	0.70 (0.50–0.97)
				65–74	136	0.61 (0.40–0.63)
				≥75	32	0.65 (0.25–1.68)
CM 9LA [51]	Nivolumab + Ipilimumab + 2 courses chemotherapy vs. standard platinum doublet chemotherapy	1L	NSCLC, PD-L1 any	<65	354	0.64 (0.5–0.82)
				65–74	295	0.78 (0.59–1.02)
				≥75	70	1.04 (0.63–1.72)

Abbreviations. CM: CheckMate; KN: KEYNOTE; 1L: first line; 2L: second line; 3L: third line; vs.: versus.

**Table 2:** irAEs of special interest in elderly people treated with PD1/PD-L1 inhibitors.

Ref	Drug	Setting	Age	Histology	PD-L1 TPS (%)	Best Response	irAEs	irAE Outcome	irAE Treatment	ICIs therapy
Nakako S, 2022 [144]	Pembrolizumab + Chemotherapy	NR	72	NSCLC	NR	NR	Delayed G4 neutropenia at 92 and 118 days after pembrolizumab discontinuation	Resolved	SC	Discontinued
Dhenin A, 2019 [153]	Pembrolizumab	1L	79	ADC	100	CR	Rash, pericarditis, colitis and myasthenia gravis	Resolved	SC	Resumed
Cham J, 2021 [154]	Durvalumab	Post-CRT	72	ADC	80	NE	Myocarditis, myasthenia gravis, and myositis	Death	SC	Discontinued
Strickley JD, 2019 [135]	Nivolumab	2L	87	NSCLC	NR	SD	Lichen Planus Pemphigoides	Resolved	SC	Discontinued
Gracia-Cazaña T [136]	Nivolumab	NR	78	SCC	NR	PD	Stevens-Johnson syndrome	Resolved	SC	Discontinued
Kim YE, 2019 [137]	Nivolumab	NR	74	NSCLC	NR	NR	Psoriasiform dermatitis	Resolved	Etanercept + Methotrexate	Discontinued
Lopez AT, 2018 [138]	Nivolumab	1L	72	SCC	0	NR	Bollous pemphigoid	Resolved	SC	Discontinued
Cosimati A, 2020 [139]	Pembrolizumab	1L	77	ADC	55	SD	Bollous pemphigoid	Resolved	SC + doxycycline, nicotinamide, and clobetasol propionate	Discontinued
Muto Y, 2020 [140]	Pembrolizumab	NR	84	NSCLC	≥50%	CR	Bollous pemphigoid	Resolved	SC	Discontinued
Yoshikawa Y, 2021 [141]	Nivolumab	1L	75	NSCLC	NR	NR	Secondary sclerosing cholangitis	Death	SC + mycophenolate mofetil	Discontinued
Hamoir C, 2018 [142]	Nivolumab	2L	71	NSCLC	NR	PD	Intrahepatic cholangitis	Resolved	SC + Ursodeoxycholic acid	Discontinued
Gelsomino F, 2017 [143]	Nivolumab	NR	79	NSCLC	NR	PD	Cholangitis	Resolved	SC + Ursodeoxycholic acid	Discontinued
Nakamura M, 2020 [145]	Durvalumab	Post-CRT	79	ADC	NR	NR	Immune-related hepatitis	Resolved	SC	Discontinued
Miyauchi M, 2020 [146]	Nivolumab	NR	79	NSCLC	NR	NR	Type 1 diabetes mellitus	Ongoing	Insulin	Discontinued
Seo JH, 2022 [147]	Nivolumab	2L	74	ADC	40	SD	Type 1 diabetes mellitus	Ongoing	Insulin	Discontinued
Matsuura N, 2018 [148]	Nivolumab	2L	78	NSCLC	NR	NR	Type 1 diabetes mellitus	Ongoing	Insulin	Resumed
Kajal S, 2021 [149]	Nivolumab	3L	84	ADC	NR	NR	Hypophysitis	Improved	SC	Resumed
Galliazzo S, 2022 [150]	Nivolumab	NR	74	SCC	NR	NR	Primary adrenal insufficiency	Improved	Glucocorticoid + mineralocorticoid	NR
Kim J, 2019 [151]	Nivolumab	2L	76	NSCLC	75	PR	Myasthenia Gravis and Myopathy	Improved	SC + ivIg	Discontinued
Hasegawa Y, 2017 [152]	Nivolumab	3L	76	ADC	NR	PD	Myasthenia gravis	Improved	SC + ivIg	Discontinued
Tan JL, 2019 [155]	Nivolumab	2L	74	ADC	NR	NR	Myocarditis and complete atrioventricular block	Improved	SC	Discontinued
Semper H, 2016 [156]	Nivolumab	2L	75	ADC	0	PR	Myocarditis	Improved	SC	Discontinued
Katsume Y, 2018 [157]	Pembrolizumab	2L	73	NSCLC	NR	NR	Myocarditis and complete atrioventricular block	Improved	SC	Discontinued
Samejima Y, 2020 [158]	Nivolumab	2L	79	SCC	NR	PR	Severe Heart Failure	Improved	Diuretics,	Discontinued
Berry EC, 2022 [159]	Atezolizumab	2L	83	ADC	NR	NR	Arteritic Anterior Ischemic Optic Neuropathy	NE	SC + Methotrexate	Discontinued
Gupta S, 2020 [160]	Durvalumab	Post-CRT	80	NSCLC	NR	NR	Vasculitis (blue toe syndrome)	Improved	SC	Discontinued
Suwa S, 2022 [161]	Atezolizumab	2L	76	NSCLC	NR	NR	Vogt–Koyanagi–Harada Disease-like Uveitis	Improved	SC	Discontinued
Shiral AC, 2016 [162]	Nivolumab	4L	73	SCC	NR	NR	Acute Interstitial Nephritis	Improved	SC	Resumed
	Nivolumab	4L	78	ADC	NR	NR	Acute Interstitial Nephritis	Resolved	SC	Discontinued
Taki T, 2020 [134]	Pembrolizumab	2L	75	ADC	25–49	PR	Tubulointerstitial Nephritis	Improved	SC	Discontinued
Takahashi N, 2018 [163]	Nivolumab	2L	74	ADC	NR	PR	Goodpasture’s disease	Death	SC + plasma exchange	Discontinued
Narumi Y, 2018 [164]	Nivolumab	2L	75	SCC	NR	PD	Neuromyelitis optica spectrum disorder	Improved	SC + plasma exchange	Discontinued
Makri OE, 2022 [165]	Pembrolizumab	2L	76	NSCLC	NR	NR	Optic Neuritis	Improved	SC	Discontinued
Richard K, 2017 [166]	Nivolumab	2L	74	NSCLC	NR	NR	Encephalitis	Improved	SC	Discontinued
Lou Y, 2019 [167]	Nivolumab	2L	73	ADC	NR	PR	Recurrent Hypereosinophilia	Improved	NR	Discontinued
Turgeman I, 2017 [168]	Nivolumab	3L	74	ADC	NR	PR	Severe Neutropenia	Improved	SC	Discontinued
Tabchi S, 2016 [169]	Nivolumab	2L	74	ADC	NR	NR	Severe agranulocytosis	NR	SC + ivIg	Discontinued
Naqash AR, 2019 [170]	Pembrolizumab	2L	74	ADC	50	PR	Recurrent febrile neutropenia	Death	SC	Discontinued
Karakas Y, 2017 [171]	Nivolumab	2L	78	NSCLC	NR	PD	Immune Thrombocytopenia	Improved	SC	Discontinued
Okawa S, 2019 [172]	Pembrolizumab	1L	78	SCC	>50	PR	Autoimmune hemolytic anemia and hemophagocytic lymphohistiocytosis	Improved	SC	Discontinued

Abbreviations. 1L: first line; 2L: second line; 3L: third line; 4L: fourth line; ADC: adenocarcinoma; SCC: squamous cell carcinoma; NE: not evaluable; NR: not reported; CR: complete response; SD: stable disease; PD: progressive disease; CRT: chemoradiotherapy; SC: systemic corticosteroid; ivIg: intravenous immunoglobulin.

## Data Availability

Not applicable.
